# The deubiquitinating enzyme USP37 enhances CHK1 activity to promote the cellular response to replication stress

**DOI:** 10.1016/j.jbc.2021.101184

**Published:** 2021-09-10

**Authors:** Benjamin R. Stromberg, Mayank Singh, Adrian E. Torres, Amy C. Burrows, Debjani Pal, Christine Insinna, Yosup Rhee, Andrew S. Dickson, Christopher J. Westlake, Matthew K. Summers

**Affiliations:** 1 Department of Radiation Oncology, Arthur G James Comprehensive Cancer Center and Richard L. Solove Research Institute, The Ohio State University, Columbus, Ohio, USA; 2 Biomedical Sciences Graduate Program, The Ohio State University, Columbus, Ohio, USA; 3 Department of Cancer Biology, Lerner Research Institute, Cleveland Clinic, Cleveland, Ohio, USA; 4 NCI-Frederick National Laboratory, Laboratory of Cellular and Developmental Signaling, Frederick, Maryland, USA

**Keywords:** cell cycle, checkpoint control, DNA replication, DNA damage response, deubiquitination, ubiquitination, CHK1, USP37, βTRCP, β-transducing repeat–containing protein, APC/C, anaphase-promoting complex/Cyclosome, APH, aphidicolin, ATR, ataxia telangiectasia and Rad3-related, BSA, bovine serum albumin, APC/C^CDH1^, cyclosome in complex with the activator CDH1, CHK1, checkpoint kinase 1, CHX, cycloheximide, DDR, DNA damage response, DUB, deubiquitinating enzyme, GST, glutathione-*S*-transferase, HDAC6, histone deacetylase 6, HU, hydroxyurea, PBST, PBS with Tween-20, SCF, SKP1–CUL1–F-box, SCF^βTRCP^, SKP1–CUL1–F-box in complex with the β-transducing repeat–containing protein, TBST, Tris-buffered saline with Tween-20, Ub, ubiquitin

## Abstract

The deubiquitinating enzyme USP37 is known to contribute to timely onset of S phase and progression of mitosis. However, it is not clear if USP37 is required beyond S-phase entry despite expression and activity of USP37 peaking within S phase. We have utilized flow cytometry and microscopy to analyze populations of replicating cells labeled with thymidine analogs and monitored mitotic entry in synchronized cells to determine that USP37-depleted cells exhibited altered S-phase kinetics. Further analysis revealed that cells depleted of USP37 harbored increased levels of the replication stress and DNA damage markers γH2AX and 53BP1 in response to perturbed replication. Depletion of USP37 also reduced cellular proliferation and led to increased sensitivity to agents that induce replication stress. Underlying the increased sensitivity, we found that the checkpoint kinase 1 is destabilized in the absence of USP37, attenuating its function. We further demonstrated that USP37 deubiquitinates checkpoint kinase 1, promoting its stability. Together, our results establish that USP37 is required beyond S-phase entry to promote the efficiency and fidelity of replication. These data further define the role of USP37 in the regulation of cell proliferation and contribute to an evolving understanding of USP37 as a multifaceted regulator of genome stability.

Cells encounter a multitude of intrinsic and extrinsic barriers to accurate DNA replication. To ensure that replication is error free, eukaryotes possess a conserved checkpoint that monitors the replication process. Upon replication stress (*e.g.*, stalled replication fork, nucleotide deficiency, DNA damage), the ataxia telangiectasia and Rad3-related (ATR) kinase is recruited to DNA (1, 2). The checkpoint mediators RAD17 and CLASPIN then recruit the effector kinase checkpoint kinase 1 (CHK1), which is phosphorylated and activated by ATR at S317 and S345. In turn, CHK1 phosphorylates a number of proteins to prevent origin firing and entry into mitosis as well as promoting DNA repair (2, 3). In the absence of CHK1 recruitment and activation upon replication stress, cells maintain high levels of CDK activity, continue to fire origins, and accumulate additional damage and chromosomal abnormalities. These cells are thus highly sensitive to additional replication stress. Importantly, high levels of replication stress are associated with high rates of proliferation during early development and expression of multiple oncogenes (*e.g.*, cyclin E, c-MYC) (4, 5, 6, 7, 8, 9). Transformation thus requires that cells survive with these abnormal levels of replication stress (7). As a result, transformed cells are highly dependent on the ATR–CLASPIN–CHK1 pathway for survival and are sensitive to agents that either induce additional stress or inhibit this critical checkpoint (5, 8, 9).

Intriguingly, premature CHK1 activation may drive S-phase entry, and failure to downregulate CHK1 activation is also detrimental to cellular survival. CHK1 activity is thus tightly regulated on multiple levels including ubiquitin (Ub)-mediated degradation of key components of the replication stress response pathway. During G1 phase, the anaphase-promoting complex/cyclosome in complex with the activator CDH1 (APC/C^CDH1^) Ub ligase prevents premature accumulation of both CLASPIN and RAD17 and hence premature S-phase entry (10, 11, 12). During G2 and mitosis phases, APC/C^CDH1^ and a second Ub ligase, SKP1–CUL1–F-box (SCF) in complex with the F-box and activator protein, β-transducing repeat–containing protein (βTRCP) (SCF^βTRCP^), prevent additional CHK1 activation by triggering the destruction of RAD17 and CLASPIN, respectively (13, 14, 15). In addition, the E3 ligases, HERC2 and BRCA1, have also been demonstrated to ubiquitinate CLASPIN (16, 17, 18). Further underscoring the need for tight control of CHK1 activation, multiple deubiquitinating enzymes (DUBs), including USP7, USP9x, USP28, and USP29, enhance CHK1 activation by stabilizing CLASPIN, whereas several recent reports demonstrate that USP20 promotes CHK1 function by stabilizing both RAD17 and CLASPIN (11, 12, 16, 17, 19, 20, 21, 22). CHK1 itself is also targeted for destruction. Upon activation, CHK1 adopts an open conformation, which exposes degrons that are recognized by SCF^FBX6^ and the Cullin 4-based CULLIN–RING ligase (CRL4^CDT2^) Ub ligases (23, 24, 25, 26). Mutations that hinder ATR-mediated conversion to the open conformation promote CHK1 stability, whereas those that cause a constitutively open conformation decrease stability (23, 24, 25, 26, 27). Additional ligases, histone deacetylase 6 (HDAC6) and HUWE1, also regulate CHK1 stability (28, 29). Although it is not clear whether the interaction of CHK1 with HDAC6 and HUWE1 requires the open conformation, ubiquitination by HUWE1 targets the kinase domain, suggesting that this conformation is the preferred substrate. Similarly, loss of HDAC6 activity results in elevated and prolonged levels of phosphorylated CHK1, consistent with a failure to target open CHK1. Not surprisingly, DUB activity also plays a role in the maintenance of CHK1 levels. However, there are few examples of CHK1 stabilization by DUBs in comparison to their involvement of stabilizing proteins that direct the activation of CHK1. To date, USP7, ATAXIN-3, and USP1 have been implicated in the maintenance of CHK1 levels with USP1 acting indirectly (30, 31, 32, 33). Together, these mechanisms confine checkpoint activity to S phase, promote recovery from checkpoint activity, and prevent hyperactivation of the checkpoint.

We recently determined that, similar to CLASPIN, the expression of the DUB USP37 is primarily confined to S phase by the concerted efforts of APC/C^CDH1^ and SCF^βTRCP^, indicating roles of USP37 in the replication process (34, 35). Indeed, USP37 regulates S phase entry *via* stabilization of cyclin A and the licensing factor, CDT1 (35, 36). In the absence of USP37 replication, fork speed is also delayed (36). However, the function(s) of USP37 after S-phase entry and the biological consequences of deficiencies in USP37 function are not well characterized. Here, we describe a requirement for USP37 in the tolerance of replication stress, which is reflected in the ability of USP37 to deubiquitinate the active form of CHK1 leading to its stabilization and the maintenance of CHK1 activity.

## Results

### USP37 promotes efficient replication

We and others have previously demonstrated that USP37 regulates the timing of S-phase entry, at least in part by facilitating the stability and accumulation of cyclin A and CDT1 during G1 phase (35, 36). However, it remains unclear whether USP37 is required beyond S-phase entry. We next sought to determine whether loss of USP37 impacts S-phase progression.

**Figure 1:**
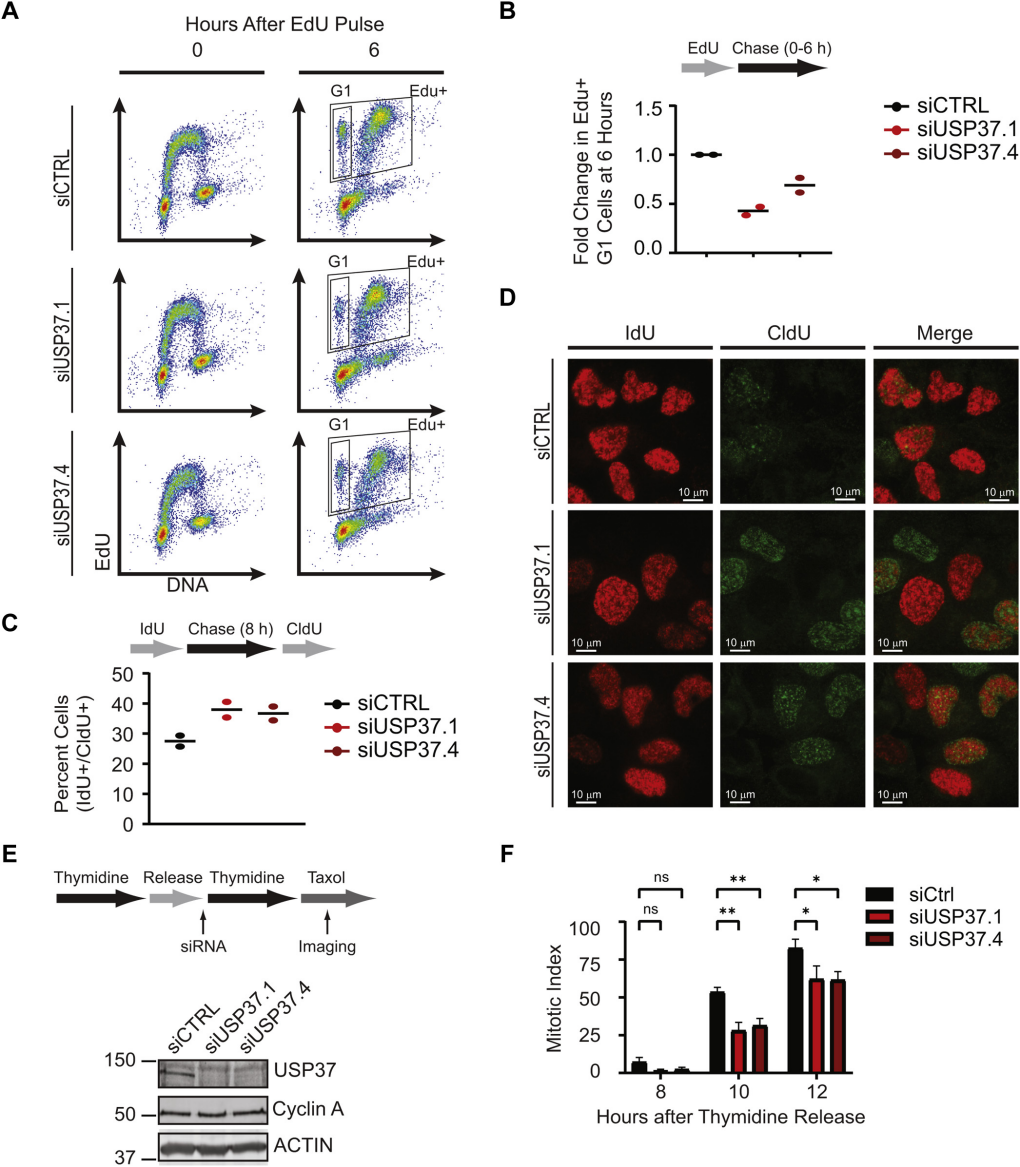
**USP37 promotes progression through S phase.***A* and *B*, analysis of cell cycle progression in the absence of USP37. *A*, representative flow cytometry analyses for an experiment. *B*, schematic representation of the experimental scheme (*top*), quantification of the fold change in EdU-positive (EdU+) HCT116 cells that had transited to G1 at 6 h after labeling (*bottom*). Individual data points for two experiments are shown. *C*, schematic representation of the experimental scheme (*top*), quantification of the percent of in IdU-positive (IdU+) U2OS cells that incorporated CldU (CldU+) 8 h later (*bottom*). Individual data points for two experiments are shown. *D*, representative images for an experiment from (*C*). *E*, schematic representation of USP37 depletion/synchronization scheme, *left*. HeLa cells, treated as in *E*, were analyzed by immunoblot for the indicated proteins. *F*, HeLa cells, treated as in *E*, were analyzed for mitotic entry by imaging live cells, at the indicated time points. Data were analyzed by two-way ANOVA with Holm–Sidak post-test. ∗*p* < 0.05; ∗∗*p* < 0.01.

Because nocodazole-synchronized USP37-depleted cells enter S phase with delayed kinetics, we sought to analyze the effects of USP37 depletion in otherwise unperturbed cells that had already entered S phase. We thus labeled asynchronous populations of control or USP37-depleted HCT116 cells with EdU, to mark replicating cells, and analyzed these cells by flow cytometry (Fig. 1, *A* and *B*). Consistent with a recent report, USP37 depletion had no gross impact on the overall cell cycle profile of asynchronous populations (Fig. 1*A*, *top panels*) (36). We then examined cell cycle progression by asking how many cells that had been in S phase (EdU+) had transited the cell cycle and now had a 2N, G1 DNA content. At 6 h after labeling, there was a ∼50% reduction in EdU+ G1 cells in the USP37-depleted populations, suggesting that USP37-depleted cells proceed slowly through S phase (Fig. 1*B*). However, a requirement for USP37 in efficient progression through mitosis has also been reported, which could delay transition of the EdU+ cells to G1 in this context (37). To confirm that delayed S-phase progression contributed to the altered cell cycle dynamics rather than mitotic defects, we performed dual labeling experiments in asynchronous U2OS cells to determine whether USP37-depleted cells indeed spend more time in S phase. Cells were labeled with IdU to mark replicating cells. After an 8-h chase, replicating cells were again identified by pulse labeling with CldU. The incorporation of CldU in the IdU+ population was analyzed to determine the fraction of cells that remained in S phase. In keeping with a role for USP37 in S-phase progression, USP37-depleted cells demonstrated a modest, but reproducible, increase in the percentage of cells remaining in S phase (IdU+, CldU+, Fig. 1, *C* and *D*).

Because cyclin A accumulation was delayed, but not abolished in USP37-depleted cells, and CDT1 is not regulated by USP37 in S phase, we reasoned that synchronizing these cells at G1/S would allow cyclin A accumulation and synchronous S-phase entry (35, 36). Given that USP37 is degraded during G2 and late mitosis/G1 by the SCF^βTRCP^ and APC/C^CDH1^ ligases, respectively, we transfected HeLa cells with control or USP37-targeting siRNAs just prior to the intervening mitosis of a double thymidine block to prevent the accumulation of USP37 during the subsequent G1 phase (Fig. 1*E*, *left panel*). Indeed, cyclin A accumulated to comparable levels in both populations by the end of the second block (Fig. 1*E*, *right panel*). If USP37 acts only to promote the timely entry into S phase, then cell cycle progression should proceed similarly in control and USP37-depleted cells from this point. After release from thymidine, we treated cells with taxol to trap cells in mitosis and monitored mitotic entry by imaging live cultures over time. USP37-depleted cells exhibited a marked delay in mitotic accumulation, suggesting that these cells were transiting slowly through S phase (Fig. 1*F*). Together, our results suggest that replication kinetics are diminished in the absence of USP37.

### USP37 loss leads to replication stress

**Figure 2:**
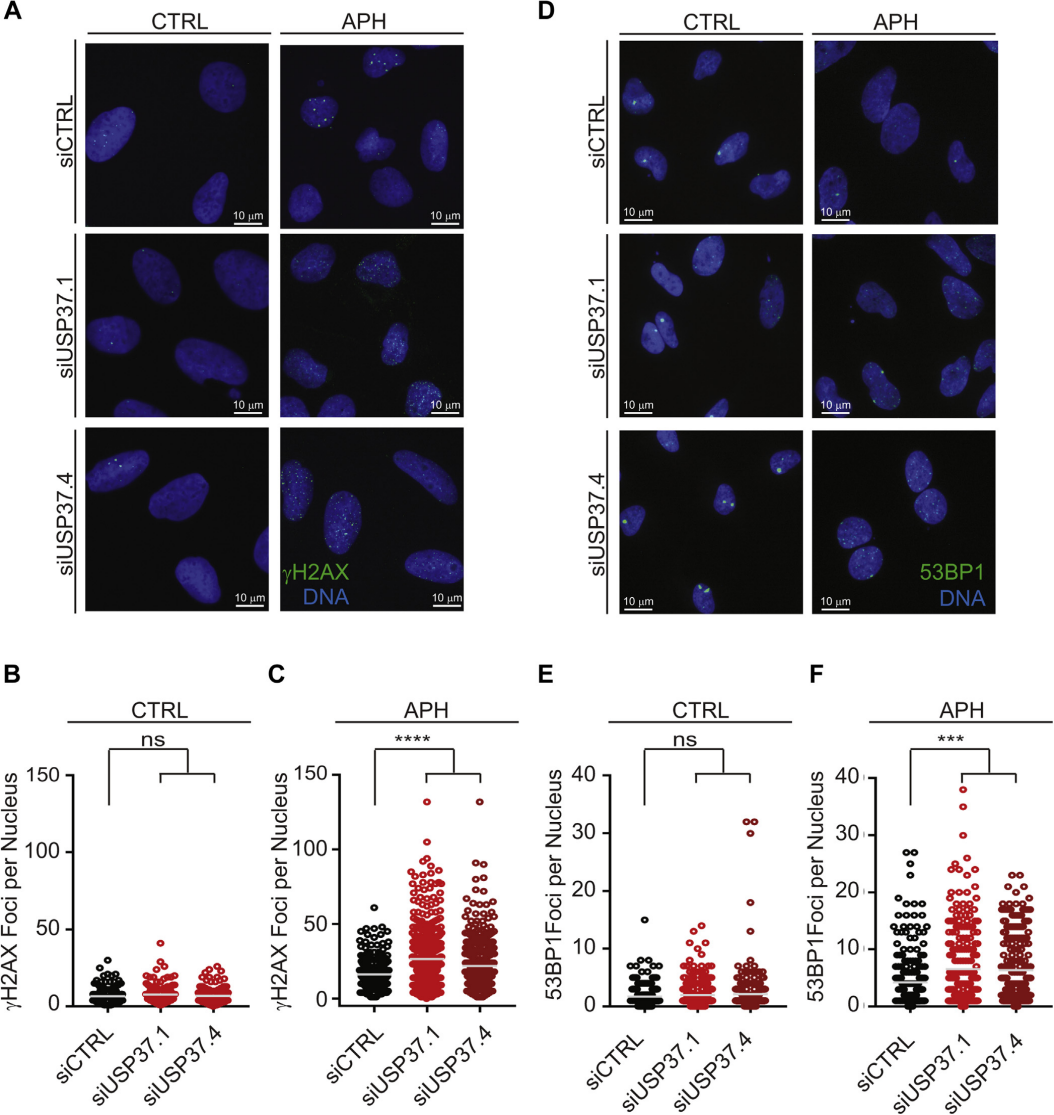
**USP37-depleted cells display increased sensitivity to replication stress.***A*, U2OS transfected with the indicated siRNAs were analyzed for the formation of γH2AX foci. *B*, quantification of the number of foci per nucleus, as in *A*, under control conditions, n > 140 cells per condition. *C*, quantification of the number of foci per nucleus, as in *A*, after treatment with 200 nM aphidicolin (APH), n > 270 cells per condition. The mean foci number is indicated. *D*, HeLa transfected with the indicated siRNAs were analyzed for the formation of 53BP1 foci. *E*, quantification of the number of foci per nucleus, as in *D*, under control conditions, n > 200 cells per condition. *F*, quantification of the number of foci per nucleus, as in *D*, after treatment with 200 nM APH, n > 200 cells per condition. The mean foci number is indicated. For all experiments, data were analyzed by one-way ANOVA with Holm–Sidak post-test. ∗∗∗*p* < 0.001; ∗∗∗∗*p* < 0.0001.

**Figure 3:**
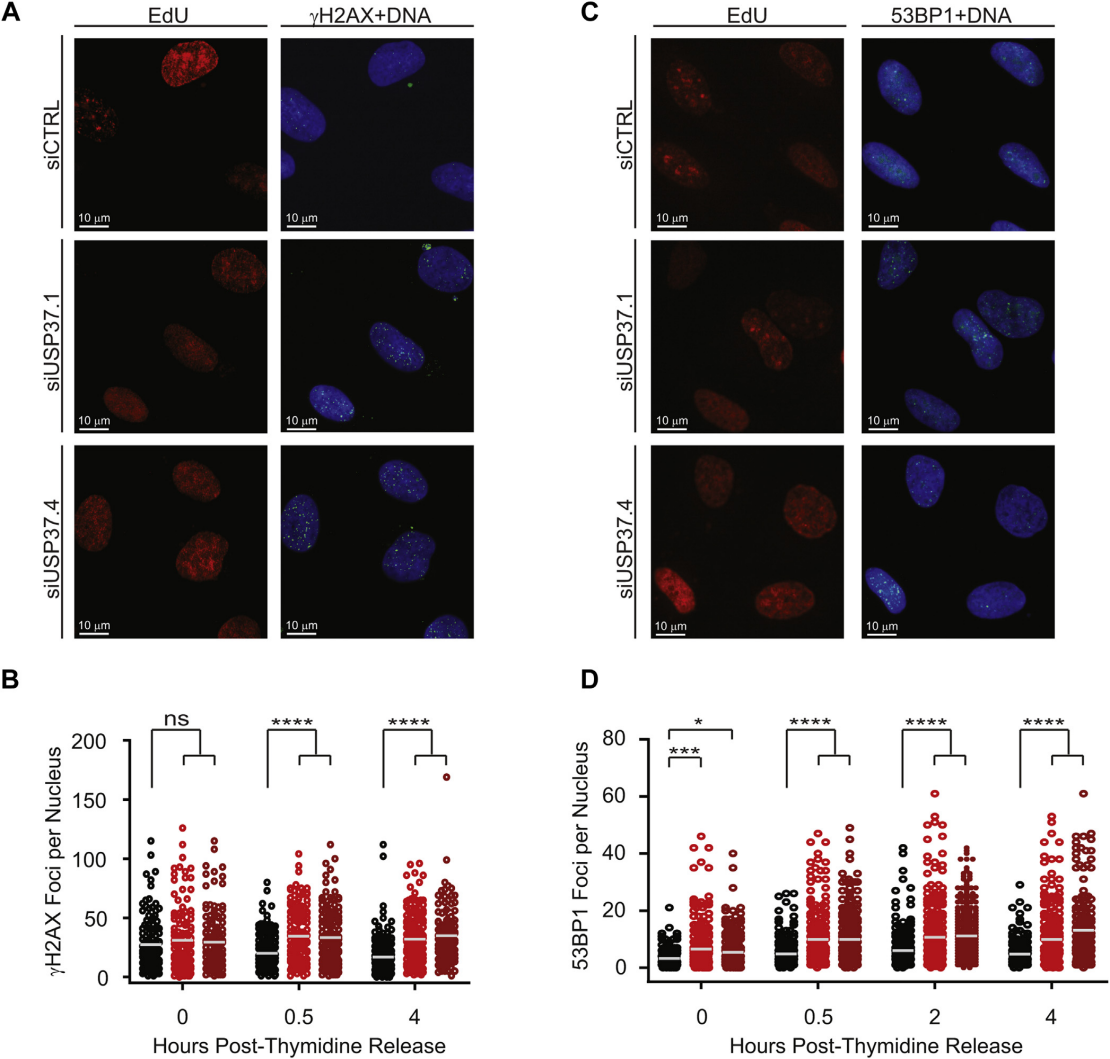
**USP37-depleted cells display increased DNA damage markers during perturbed replication.***A*, U2OS cells transfected with the indicated siRNAs (Fig. 1, *A* and *B*) were analyzed for the formation of γH2AX foci. Images from 4 h after thymidine release are shown. *B*, quantification of the number of foci per nucleus cells, as in *A*, n > 120 cells per condition. The mean foci number is indicated. *C*, HeLa transfected with the indicated siRNAs (Fig. 1, *A* and *B*) were analyzed for the formation of 53BP1 foci. Images from 4 h after thymidine release are shown. *D*, quantification of the number of foci per nucleus cells, as in *D*, n > 150 cells per condition. The mean foci number is indicated. For all experiments, data were analyzed by one-way ANOVA with Holm–Sidak post-test. ∗*p* < 0.05, ∗∗∗*p* < 0.001, and ∗∗∗∗*p* < 0.0001.

Our results thus far indicate that USP37 promotes efficient progression of the replication process. We noticed that the delays in progression through S phase appeared to be more pronounced in the synchronized population. While synchronization *via* thymidine blocks is widely used to study cell cycle progression and generally considered innocuous, it does cause replication stress (38). Thus, our observation suggested that USP37-depleted cells may be undergoing and/or sensitive to replication stress. We thus examined the impact of USP37 loss on the emergence of DNA damage in replicating cells. Examination of γH2AX (Fig. 2, *A* and *B*) or 53BP1 (Fig. 2, *E* and *F*) foci in USP37-depleted cells did not indicate significant increases in replication stress/DNA damage in U2OS and HeLa cells. However, upon treatment with low doses of the replication poison aphidicolin (APH), there is a significant increase in the number of foci for both these markers in the absence of USP37 (Fig. 2, *A*, *C*, *D*, and *F*). We next depleted USP37 from synchronized cells as in Figure 1*E* and analyzed replication stress/DNA damage as aforementioned. Consistent with previous reports, double-thymidine blocked cells exhibited increased γH2AX foci, which were similar between control and USP37-depleted cells (Fig. 3, *A* and *B*) (38). Indeed, labeling cells with EdU demonstrated that USP37-depleted cells initiate replication upon release from the thymidine block comparably to control-depleted cells (Fig. S1), consistent with the accumulation of cyclin A in synchronized cells (as in Fig. 1*E*). However, while control populations exhibited reduced γH2AX foci per nucleus as cells began replication, the number of foci in USP37-depleted cells remained elevated. These analyses revealed that USP37-depleted cells exhibit increased levels of DNA damage in early S phase upon release from thymidine. Similarly, double-thymidine blocked cells also exhibited elevated levels of 53BP1 foci. Upon release from the thymidine block, the number of 53BP1 foci in USP37-depleted cells increased as cells underwent replication, in contrast to control cells (Fig. 3, *C* and *D*). Together, these data suggest that USP37 promotes proper progression through S phase and that the loss of USP37 leads to enhanced sensitivity to replication stress.

### USP37 promotes replication stress tolerance

**Figure 4:**
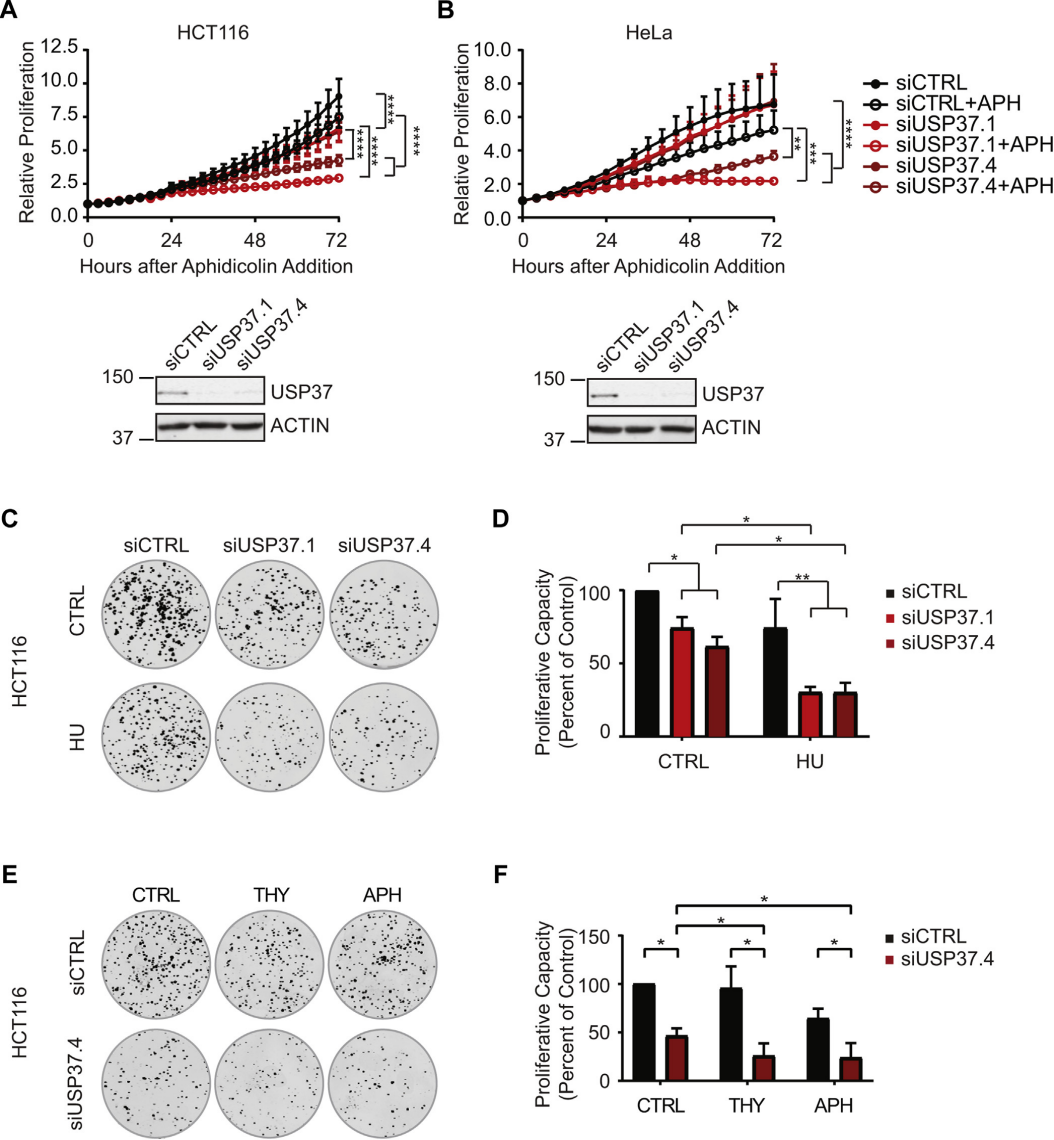
**USP37 promotes proliferation and tolerance of replication stress.***A*, *upper panel*, cell proliferation (normalized to *t* = 0) was analyzed in HCT116-H2BGFP cells transfected with the indicated siRNAs ±100 nM aphidicolin (APH). Data from two independent experiments, performed in triplicate. Mean and SD are indicated. Data were analyzed by two-way ANOVA with Tukey's post-test; ∗∗∗∗*p* < 0.0001. *Lower panels*, cell lysates were analyzed by immunoblot with the indicated antibodies. *B*, *upper panel*, cell proliferation (normalized to *t* = 0) was analyzed in HeLa cells transfected with the indicated siRNAs ±200 nM APH. Data from two independent experiments were performed in triplicate. Mean and SD are indicated. Data were analyzed by two-way ANOVA with Tukey's post-test; ∗∗*p* < 0.01, ∗∗∗*p* < 0.001, and ∗∗∗∗*p* < 0.0001. *Lower panels*, cell lysates were analyzed by immunoblot with the indicated antibodies. *C* and *D*, colony formation of HCT116 cells transfected with the indicated siRNAs ± treatment with 1 mM hydroxyurea (HU) for 18 h. *C*, representative images. *D*, quantification of data as in (*C*), normalized to control, no drug. Data from two independent experiments were performed in triplicate. Mean and SD are indicated. Data were analyzed by two-way ANOVA with Tukey's post-test; ∗*p* < 0.05 and ∗∗*p* < 0.01. *E* and *F*, colony formation of HCT116 cells transfected with the indicated siRNAs ± treatment with 1 mM HU, 200 mM thymidine (THY), or 7 μM APH for 18 h. *E*, representative images. *F*, quantification of data as in (*E*), normalized to control, no drug. Data from three independent experiments, performed in triplicate, are shown. Mean and SD are indicated. Data were analyzed by two-way ANOVA with Holm–Sidak post-test; ∗*p* < 0.05.

To gain further insight into the biological impact of the increased sensitivity to replication stress upon depletion of USP37, we examined the proliferation of cells with and without replication stress. Real-time proliferation analyses were performed on USP37-depleted HCT116 and HeLa cells with and without addition of APH to induce stress. In both cases, USP37-depleted cells proliferated more slowly than the control populations, although this effect was less pronounced in HeLa cells (Fig. 4, *A* and *B*). Moreover, in line with the increased levels of replication stress, USP37-depleted cells in both cell lines showed significant loss of proliferation upon induction of replication stress in comparison to both control cells undergoing stress and USP37-depleted cells in the absence of induced stress. Further analysis revealed a dose-dependent response in HCT116 cells (Fig. S2, *A*–*C*). To extend these findings, we examined the effects of USP37 depletion in multiple cell lines. Similar sensitivity was observed in a third cell line, H1299, with hydroxyurea (HU) as the stress-inducing agent (Fig. S2*D*). Two additional cell lines, MCF7 and MDA-MB-468, exhibited clearly diminished proliferation upon USP37 depletion in the absence of drug with MCF7 exhibiting marked sensitivity (Fig. S2, *E* and *F*). To further examine the effects of USP37 depletion, we transfected H1299 cells with an siRNA targeting the USP37 3′UTR along with control vector or USP37 complementary DNA. Targeting the 3′UTR led to a growth defect similar to the other oligos that was significantly improved by reintroduction of USP37 (Fig. S2, *G* and *H*). We then examined the impact of USP37 depletion on the long-term proliferative capacity of cells. Consistent with our kinetic growth analyses, depletion of USP37 caused a reduction in clonogenic growth in HCT116 (Fig. 4, *C* and *D*) and H1299 (Fig. S3, *A* and *B*) cells. The growth defect was significantly exacerbated by the induction of replication stress by HU in comparison to both USP37-depleted cell proliferation in the absence of HU and control cells treated with HU. We obtained analogous results in H1299 cells expressing USP37-targeting shRNA that was rescued by reintroduction of USP37 (Fig. S3, *C* and *D*). Similar results were also obtained in U2OS cells with siRNA targeting USP37 (Fig. S3, *E* and *F*). To gain additional insight into the impact of USP37 depletion on the sensitivity to replication stress, we also treated control or siUSP37-transfected HCT116 and U2OS cells with thymidine or APH to induce replication stress and examined colony-forming potential. USP37-depleted HCT116 cells displayed significant loss of proliferation upon induction of replication stress by both agents, whereas control cells exhibited significant sensitivity only to APH, although to a lesser degree than the USP37-depleted cells (Fig. 4, *E* and *F*). U2OS cells behaved similarly. Control U2OS cells were modestly, but not significantly, sensitive to replication stress induced by all three agents, whereas USP37-depleted cells were significantly sensitive to both HU and APH. And, although it did not reach statistical significance, the colony formation capacity of USP37-depleted U2OS cells upon exposure to thymidine was dramatically diminished relative to control-depleted cells (Fig. S3, *G* and *H*).

We next extended our analyses of the effect of USP37 depletion on growth beyond cell lines and depleted Usp37 in zebrafish embryos using morpholino technology. A translation-blocking morpholino efficiently depleted Usp37 protein in embryos (Fig. S5*A*). In keeping with our cell line data, Usp37-deficient embryos exhibited a survival deficit, along with increased cellular death that was exacerbated by replication stress induced by HU (Fig. S5, *B*–*D*). Depletion of Usp37 in embryos also resulted in developmental defects that manifested as a body curvature phenotype, which was significantly enhanced by replication stress (Fig. S5, *C*, *E*, and *F*). Notably, increased body curvature has been associated with genomic instability and sensitivity to replication stress/DNA damage in developing zebrafish (39, 40, 41, 42, 43). Together, our results indicate that USP37-deficient cells are generally sensitive to replication stress.

### UPS37 promotes CHK1 activity

**Figure 5:**
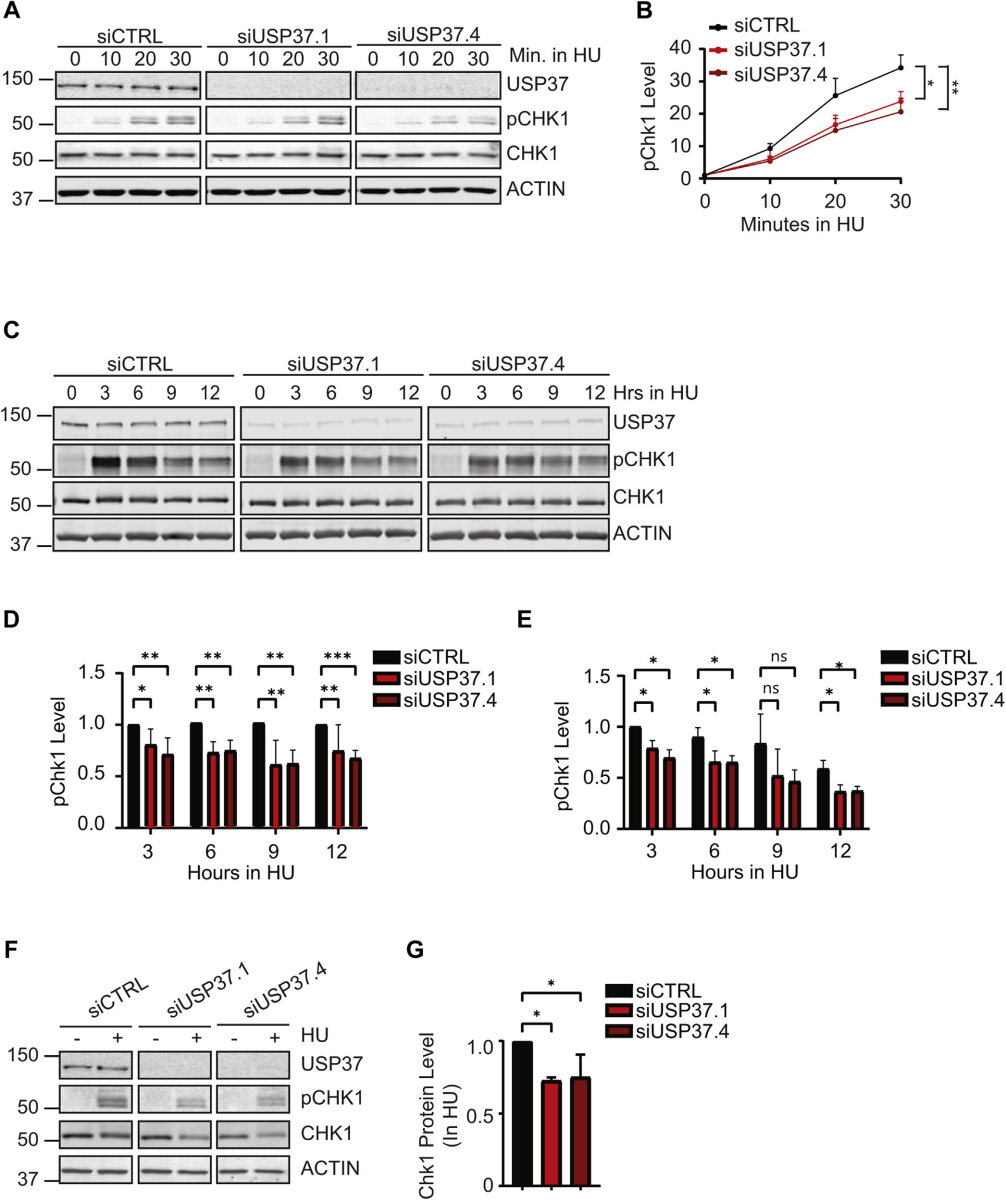
**USP37 promotes CHK1 activity.***A*, HCT116 cells were transfected with the indicated siRNAs and examined by immunoblotting at the indicated times after treatment with 10 mM hydroxyurea (HU). *B*, quantification of CHK1 phosphorylation at S345 (pCHK1), in *A* normalized to total CHK1 levels. Data from three independent experiments are shown. Mean and SD are indicated. Data were analyzed by two-way ANOVA with Holm–Sidak post-test: ∗*p* < 0.05, ∗∗*p* < 0.01. *C*, HCT116 cells transfected with the indicated siRNAs were analyzed by immunoblot after treatment with 5 mM HU. *D*, quantification of CHK1 phosphorylated at S345 (pCHK1) from cells as in *C*, normalized to total CHK1 and then to levels in siCTRL-transfected cells at each time point. Data from five independent experiments are shown. Mean and SD are shown. Data were analyzed by two-way ANOVA with Holm–Sidak post-test: ∗*p* < 0.05, ∗∗*p* < 0.01, and ∗∗∗*p* < 0.001. *E*, quantification of CHK1 phosphorylated at S345 (pCHK1) from cells as in *C*, normalized to total CHK1 and then to levels in siCTRL-transfected cells normalized to the peak pCHK1 level siCTRL at 3 h. Data from five independent experiments are shown. Mean and standard error of the mean are shown. Data were analyzed by two-way ANOVA with Holm–Sidak post-test; ∗*p* < 0.05. *F*, HCT116 cells transfected with the indicated siRNAs were analyzed by immunoblot 18 h after treatment with 2 mM HU. *G*, quantification of CHK1 protein levels after HU treatment. Data from three independent experiments are shown. Mean and SD are indicated. Data were analyzed by two-way ANOVA with Holm–Sidak post-test: ∗*p* < 0.05. CHK1, checkpoint kinase 1.

Given that USP37-depleted cells exhibit slower progression through S-phase and delayed mitotic entry, it is possible that these cells exhibit a higher basal level of replication stress and thus replication checkpoint activity. However, the fact that we do not observe evidence of DNA damage in the absence of replication stress as well as increased sensitivity to replication stress suggests that these cells may have defects in the checkpoint response. To gain insight into the status of the replication checkpoint in the absence of USP37, we examined phosphorylation of the essential replication checkpoint effector kinase CHK1 as Ser345 (pS345), which is indicative of its full activation and frequently used to measure checkpoint activation (44). We first examined the kinetics of CHK1 activation by pulsing the cells with HU. We did not observe a heightened level of basal CHK1 phosphorylation in USP37-depleted cells (Fig. 5*A*). However, although CHK1-pS345 appeared with similar kinetics and accumulated over time in both control and USP37-depleted cells, we noted that the overall levels of CHK1-pS345 were lower in the absence of USP37 (Fig. 5, *A* and *B*). The lag in CHK1 phosphorylation could result from a defect in activation of the kinase. Interestingly, the CHK1 activating phosphorylation events elicit a conformational change that not only places the kinase domain into an active conformation but also exposes degrons that prompt the destruction of active CHK1 to limit activation and promote efficient checkpoint recovery (23, 24, 25, 26). To gain further insight into the impact of USP37 on CHK1 activity, we analyzed CHK1-pS345 over an extended, 12 h, exposure to HU. CHK1-pS345 levels are attenuated at all time points (Fig. 5, *C*–*E*). However, although the levels CHK1-pS345 in USP37-depleted cells do not reach those of control cells, the levels rise and fall with similar kinetics (Fig. 5*E*). A similar reduction in pCHK1 levels was observed after prolonged replication stress that was accompanied by a 25% reduction in the level of total CHK1 (Fig. 5, *F* and *G*). Together, these data indicate that USP37-depleted cells fail to maintain active CHK1 during replication stress.

**Figure 6:**
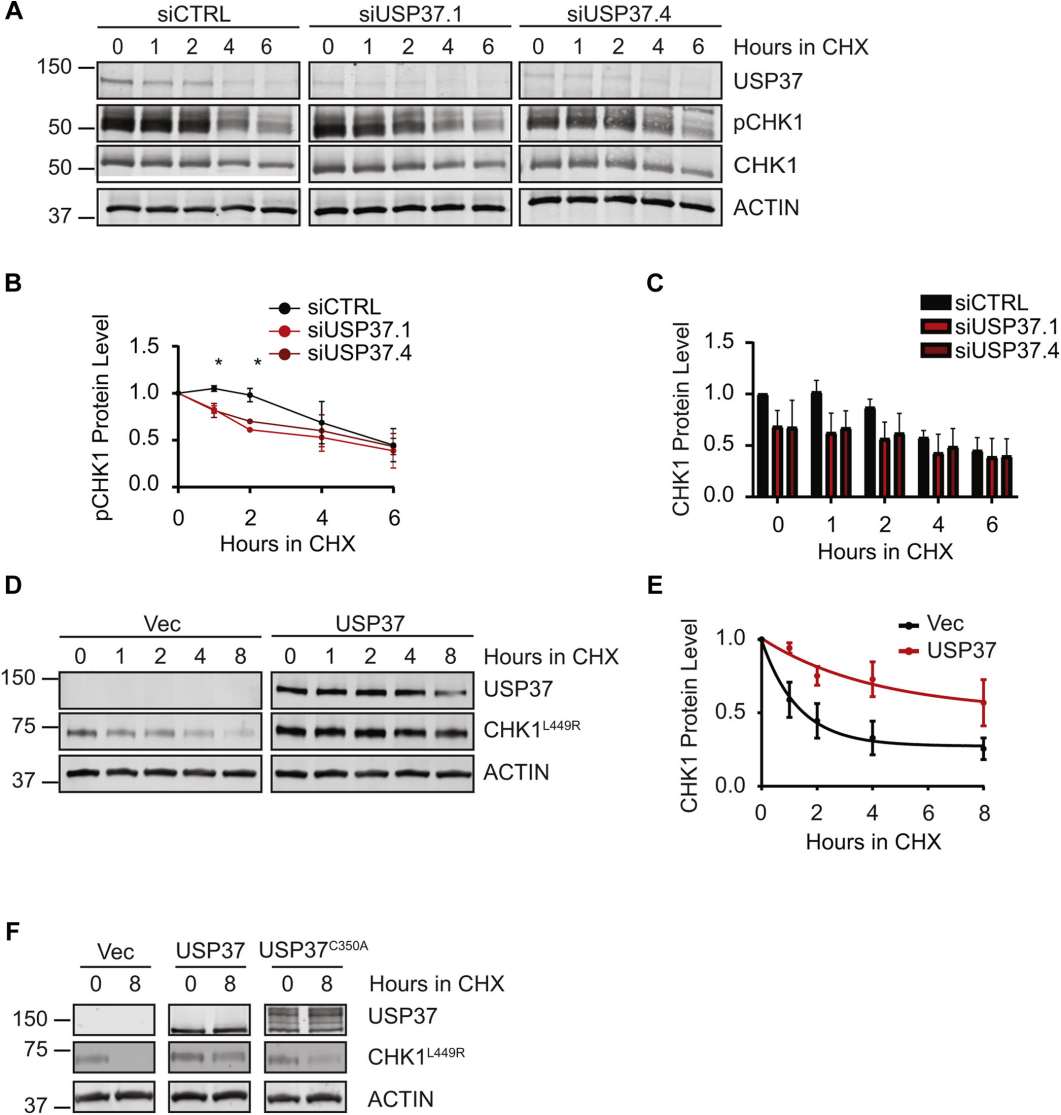
**USP37 promotes the stability of active CHK1.***A*, 293T cells were transfected with the indicated siRNAs and examined by immunoblotting after treatment with 5 mM hydroxyurea (HU) for 3 h before addition of ±100 μg/ml cycloheximide (CHX) for the indicated times. *B*, quantification of CHK1 phosphorylated at S345 (pCHK1) from cells as in *A*, normalized to total CHK1 and then to levels at the addition of CHX for each siRNA. Data from three independent experiments are shown. Mean and SD are indicated. Data were analyzed by two-way ANOVA with Holm–Sidak post-test: ∗*p* < 0.05. *C*, quantification of CHK1 from cells as in *A*, normalized to levels in siCTRL-transfected cells at the time of CHX addition. *D*, 293T cells were transfected with plasmids encoding constitutively active MYC-CHK1^L449R^ ± FLAG-USP37 and analyzed by immunoblot after treatment with 100 μg/ml CHX. *E*, quantification of data as in (*D*). Data are from three independent experiments. Mean and SD are indicated. *F*, 293T cells were transfected with plasmids encoding MYC-CHK1^L449R^ ± FLAG-USP37 or the catalytically inactive USP37^C350A^ mutant and analyzed by immunoblot after treatment with 100 μg/ml CHX. CHK1, checkpoint kinase 1.

To test the possibility that USP37 may affect CHK1 protein levels, we tested the ability of UPS37 to impact CHK1 stability. Because the active and open conformation of CHK1 is the form targeted by ligases for degradation (23, 24, 25, 26, 27), we pretreated cells with HU for 3 h before adding cycloheximide (CHX) to block translation. We found that CHK1-pS345 levels fell more rapidly in the absence of USP37 (Fig. 6, *A* and *B*). Consistent with our data in Figure 5, we observed that the levels of CHK1 were lower after treatment with HU in the absence of USP37. However, when compared with the levels in control cells at the onset of the CHX treatment, total CHK1 levels did not drop as rapidly in USP37-depleted cells (Fig. 6*C*). These data are consistent with USP37 acting on CHK1 but could also reflect a failure to activate CHK1, rendering it more stable. To gain insight into these possibilities directly, we tested the impact of USP37 on the stability of active CHK1. We took advantage of the fact that the CHK1^L449R^ mutant is in the constitutively open conformation and is predicted to be inherently unstable allowing us to test the ability of USP37 to regulate the stability of active CHK1 without replication stress and irrespective of any potential defects in CHK1 activation (45). We confirmed that CHK1^L449R^ is more unstable than the wildtype protein (Fig. S4), consistent with the stability of similar mutants reported previously (23, 24, 25, 26, 27). Cotransfection experiments in 293T cells revealed that expression of USP37 dramatically stabilizes CHK1^L449R^, extending the half-life of the protein from ∼1 to ∼2.8 h (Fig. 6, *D* and *E*). Given this impact on ectopic protein, these results suggest that USP37 acts upon CHK1 protein rather than by impacting transcription or translation. To further examine the possibility that CHK1 is a substrate of USP37, we sought to determine whether USP37 activity was required for its ability to impact CHK1 levels. Indeed, whereas expression of USP37 prevents the degradation of CHK1^L449R^ following CHX treatment, expression of the catalytically inactive USP37^C350A^ mutant failed to impact CHK1^L449R^ levels (Fig. 6*F*). Together, these data suggest that CHK1 may be a substrate of USP37.

### USP37 interacts with and deubiquitinates CHK1

**Figure 7:**
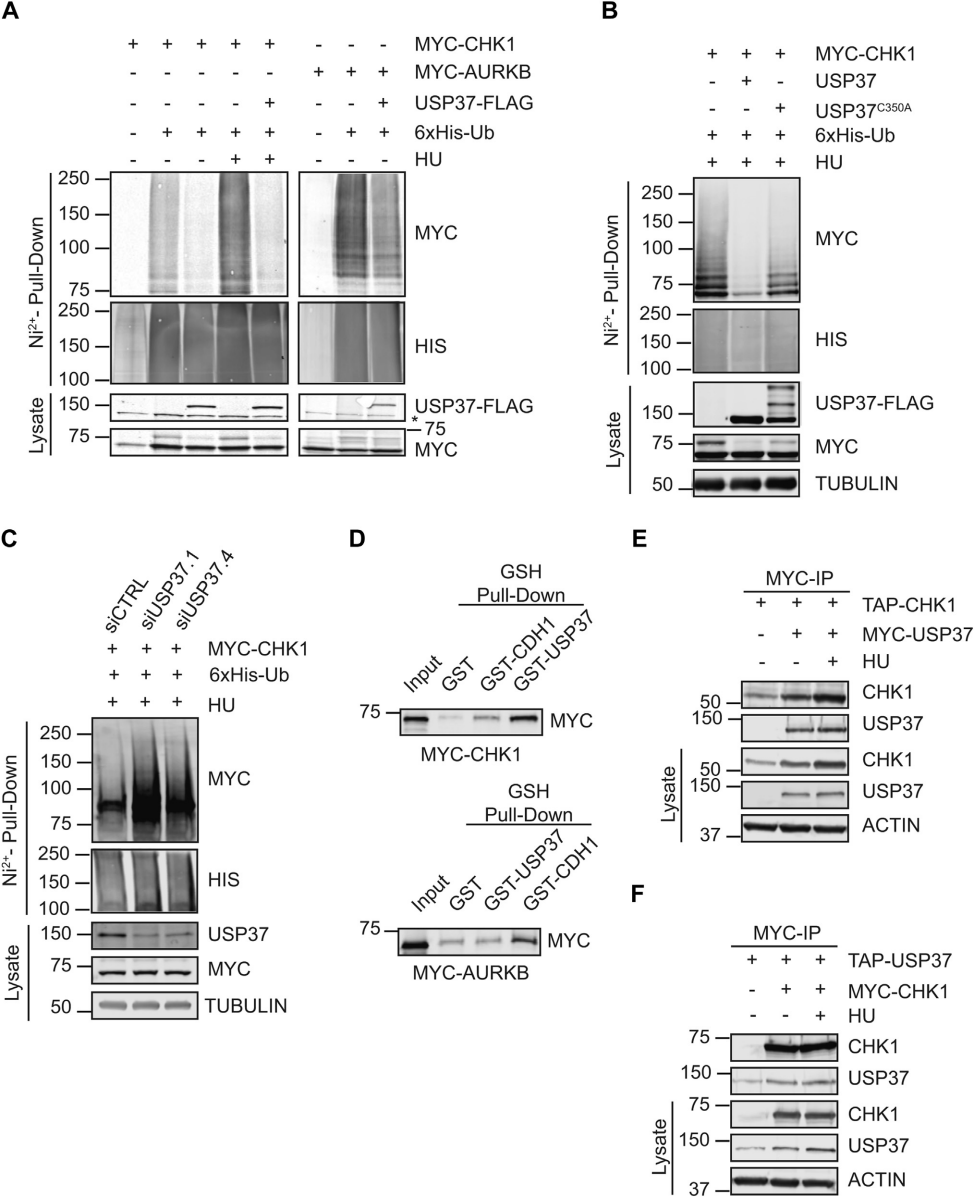
**USP37 binds and deubiquitinates CHK1.***A*, 293T cells were transfected with plasmids encoding 6xHIS-Ub and MYC-CHK1 or MYC-AURKB ± FLAG-USP37 and treated with 10 mM hydroxyurea (HU) and 10 μg/ml MG132 as indicated. Lysates and ubiquitin-conjugated proteins (Ni^2+^ pulldown) were analyzed by immunoblot. A cross-reacting band is indicated by the *asterisk*. *B*, 293T cells were transfected with plasmids encoding 6xHIS-Ub and MYC-CHK1 ± USP37-FLAG or USP37^C350A^-FLAG, as indicated, and treated with 10 mM HU and 10 μg/ml MG132 and analyzed as in *A*. *C*, 293T cells were transfected with plasmids encoding 6xHIS-Ub and MYC-CHK1 and the indicated siRNAs and treated with 10 mM HU and 10 μg/ml MG132 and analyzed as in *A*. *D*, recombinant GST-USP37 or GST was utilized as bait to capture *in vitro* translated MYC-CHK1. The captured proteins were analyzed by immunoblot. *E*, 293T cells were transfected with plasmids encoding TAP-CHK1 ± MYC-USP37 as indicated and analyzed by immunoblot after MYC immunoprecipitation. *F*, 293T cells were transfected with plasmids encoding MYC-CHK1 ± TAP-USP37 as indicated and analyzed by immunoblot after MYC immunoprecipitation. CHK1, checkpoint kinase 1.

**Figure 8:**
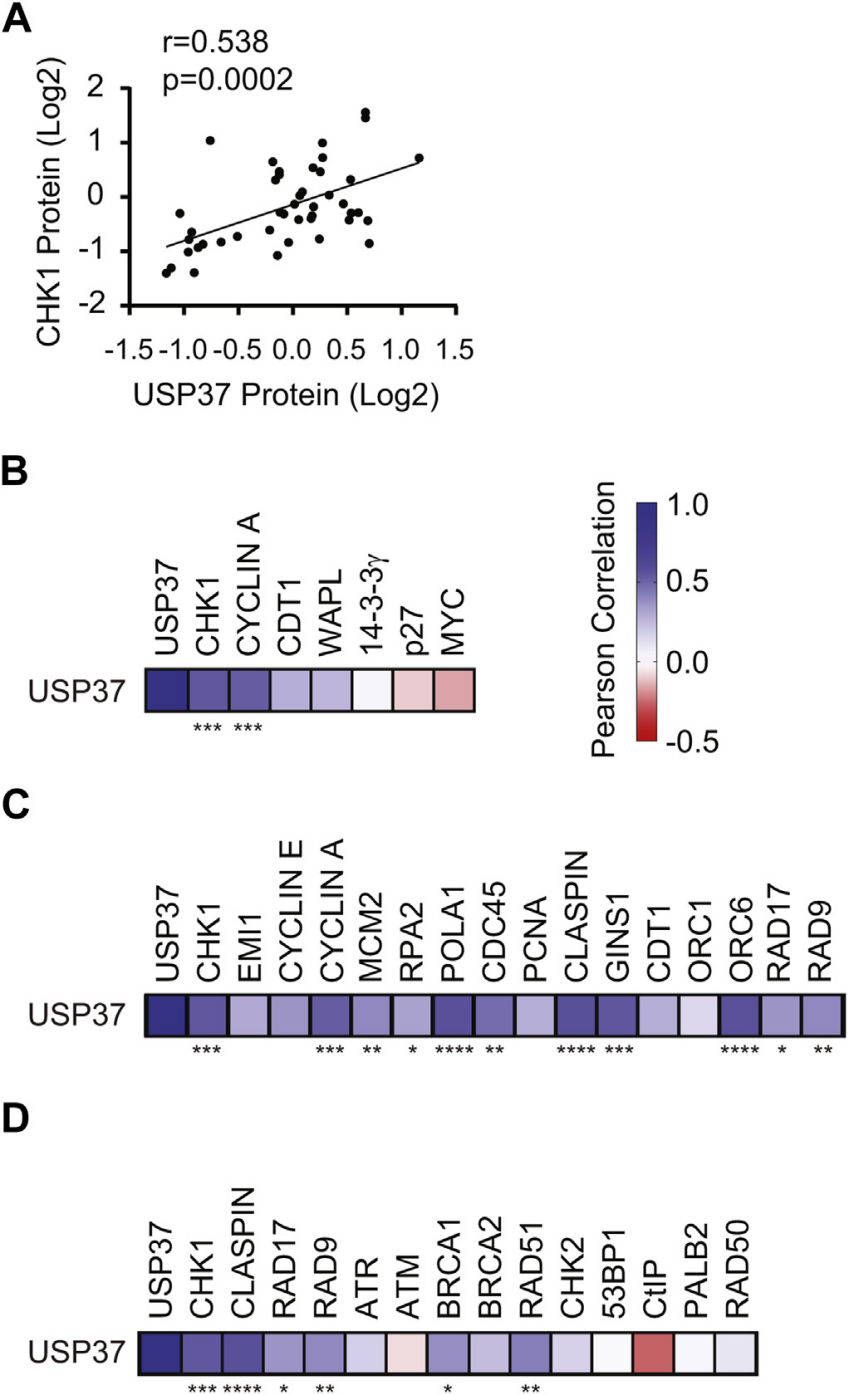
**Correlation of USP37 protein expression with substrates, replication factors, and DNA damage response and repair proteins.***A*, protein expression of USP37 and CHK1 across 44 cell lines. The *r* and *p* values determined by Pearson correlation analysis are indicated. *B*, heat map depiction of Pearson correlation analysis of protein levels of UPS37, CHK1, and reported substrates. Data from 44 cell lines are shown, except for CDT1 (n = 27) and MYC (n = 36). ∗∗∗*p* < 0.001. The color key for heat maps for *B*–*D* is shown at *right*. *C*, heat map depiction of Pearson correlation analysis of protein levels of UPS37, CHK1, and replication factors. Data from 44 cell lines are shown, except for EMI1 (n = 27), cyclin E1 (n = 18), and CDT1 (n = 27). ∗*p* < 0.05, ∗∗*p* < 0.01, ∗∗∗*p* < 0.001, and ∗∗∗∗*p* < 0.0001. *D*, heat map depiction of Pearson correlation analysis of protein levels of UPS37, CHK1, and DNA damage response and repair proteins. Data from 44 cell lines are shown, except for CtIP (n = 27) and PALB2 (n = 36). ∗*p* < 0.05, ∗∗*p* < 0.01, ∗∗∗*p* < 0.001, and ∗∗∗∗*p* < 0.0001. CHK1, checkpoint kinase 1.

Based on the aforementioned observations, we performed *in vivo* deubiquitination analyses. We coexpressed MYC-CHK1 or MYC-AURKB with 6xHIS-Ub in 293T cells with or without USP37-FLAG and analyzed the ubiquitinated proteome, purified on Ni^2+^ beads, for the presence of the MYC epitope. Consistent with the results of our stability assays, expression of USP37 resulted in a nearly complete loss of CHK1–Ub conjugates (Fig. 7*A*). The activity of USP37 toward CHK1 was specific as there were no impacts to bulk Ub conjugates or to Ub conjugates of the non-USP37–interacting kinase AURKB. To confirm that USP37 activity is required for the deubiquitination of CHK1, we performed similar experiments using the inactive USP37^C350A^ catalytic site mutant. Consistent with our results in the stability assays, the catalytically dead USP37 was unable to impact CHK1–Ub conjugates (Fig. 7*B*). In contrast to these results, depletion of USP37 led to an increase in the presence of CHK1–Ub conjugates (Fig. 7*C*). These results prompted us to test whether USP37 and CHK1 interact. In an *in vitro* binding assay, recombinant glutathione-*S*-transferase (GST)-USP37 was able to interact with *in vitro* translated CHK1, but not the AURKB kinase, whereas GST-CDH1 was able to efficiently interact with its known substrate AURKB, indicating that the protein was correctly folded and suggesting that the USP37–CHK1 interaction was specific (Fig. 7*D*). CHK1 also interacted with GST-CDH1, which we recently determined to be a substrate of the kinase, further suggesting that the *in vitro* binding interaction was valid and specific (46). Similarly, immunoprecipitation of USP37 and CHK1 proteins after coexpression in 293T cells revealed that the proteins bind *in vivo* as well (Fig. 7, *E* and *F*). Together, these results identify CHK1 as a substrate of USP37. To further explore this possibility, we examined a recent proteomic analysis of numerous cell lines (47). Among the lines tested, 44 have data for USP37 expression. Across these lines, there is a positive correlation between the protein levels of CHK1 and USP37 (Fig. 8*A*). Moreover, CHK1 shows a stronger correlation with USP37 levels across these cell lines than the majority of other reported substrates do (Fig. 8*B*) (35, 36, 37, 48, 49, 50). Only cyclin A showed a similar and significant correlation to USP37. Because cyclin A, CHK1, and USP37 are all involved in S-phase progression, we analyzed the correlation between USP37 levels and a panel of proteins involved in replication. USP37 showed varying degrees of correlation with the replication factors but did not show significant correlation with all of them indicating that the correlation with CHK1 is not simply because of similar expression patterns (Fig. 8*B*). Because USP37 has been implicated in the DNA damage response (DDR), we similarly compared its expression to a panel of proteins involved in various aspects of the DDR (51). As with the replication factors, USP37 did not show strong correlations with the majority of DDR components. However, it did show significant correlation with proteins involved in the replication stress response (Fig. 8*C*).

## Discussion

**Figure 9:**
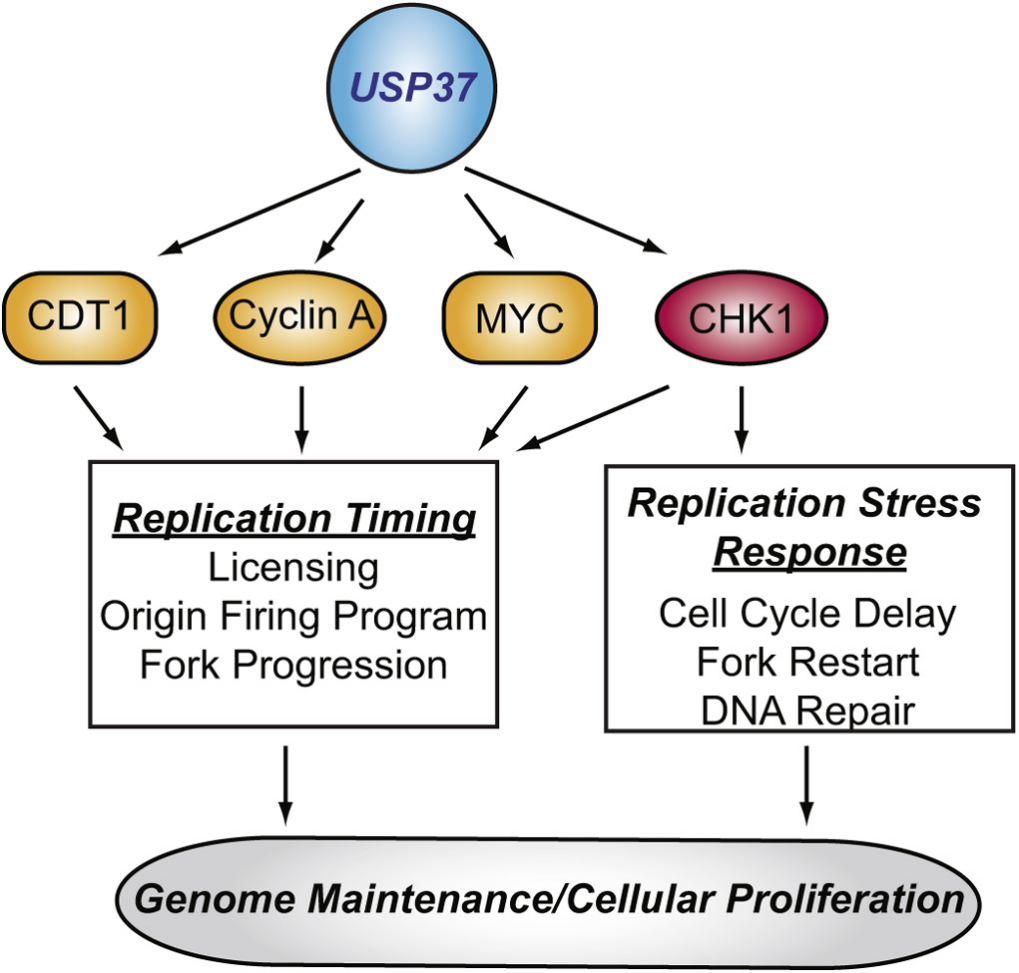
**Current model of the role of USP37 in the control of replication.** USP37 is known to regulate the timing of S phase by stabilizing factors involved in licensing (CDT1) and origin firing and fork progression (cyclin A, MYC). By promoting CHK1 stability and activity, USP37 also strengthens the control of origin firing and fork progression, while enhancing the response to perturbations in the replication process. See text for additional details. CHK1, checkpoint kinase 1.

Here, we report the identification of CHK1 as a USP37 substrate. USP37 and CHK1 interact *in vitro* and *in vivo*. Expression of USP37 promotes steady-state levels of CHK1, decreases CHK1–Ub conjugates, stabilizes an active and unstable CHK1^L449R^ mutant, and protein levels correlate in cell lines. In the absence of USP37, CHK1 stability is compromised, and cells mount an attenuated activation of CHK1. In keeping with the peak of USP37 expression and activity during late G1/S phase, CHK1 joins the growing number of USP37 substrates (including cyclin A, CDT1, and c-MYC) that are involved in the replication process and further implicates USP37 in the control of cell growth and the regulation of genome integrity (Fig. 9) (35, 36, 49). Notably, while our data demonstrate the ability of USP37 to directly regulate CHK1 activity by stabilizing the active form of the kinase, they do not exclude the possibility that USP37 may also regulate the ability of CHK1 to be activated by ATR. In support of this possibility, we have detected an interaction between USP37 and the upstream effector molecule CLASPIN (A. C. B. and M. K. S., unpublished results). Although the meaning of this interaction in terms of CHK1 activity will require additional studies, our finding that USP37 is required for full CHK1 activity provide new insight into the functions of this DUB in both cell and cancer biology.

The recent identification of CDT1 as a substrate of USP37 revealed that elevated USP37 could enhance minichromosome maintenance protein complex protein loading onto chromatin, consistent with its ability to promote S phase (35, 36). However, unexpectedly, depletion of USP37 was found to decrease replication fork speed and increase the number of replication origins fired. These observations are not anticipated for loss of CDT1, which would lead to fewer licensed origins and hence fewer origins capable of firing, a situation that permits increased fork speeds. In contrast, these observations are consistent with diminished CHK1 activity. CHK1 limits the number of forks that fire, and the increased number of firing origins observed in the absence of CHK1 function causes a delay in replication fork speed as replication factors and nucleotides become limiting (52, 53, 54). Delayed fork speeds in CHK1-inhibited cells are not associated with delayed S-phase progression, in contrast to our observations (53). However, the absence of CHK1 activity because of the use of inhibitors in these studies may mask the consequences of delayed progression. In contrast, slowing of replication fork rates has been associated with delayed cell cycle progression in other studies (55, 56). Notably, depletion of the replisome progression complex component AND-1, which also weakens CHK1 activity, attenuates fork speeds as well as delaying progression through S phase (57, 58). Our data, together with this previous work, suggest that regulation of CHK1 by USP37 promotes proper replication dynamics. However, we do not exclude the likely contribution of other yet to be identified USP37 substrates in the control of replication kinetics.

Alterations in replication dynamics are associated with replication stress. Thus, it was somewhat unexpected that USP37-depleted cells did not exhibit signs of replication stress/DNA damage. However, similar alterations in replication kinetics and cell cycle progression without detection of classical markers of replication stress or the replication stress response have been reported (55). Consistent with the idea that the USP37-depleted cells are undergoing replication stress, these cells showed enhanced induction of γH2AX and 53BP1 foci after treatment with APH along with loss of proliferative capacity upon exposure to multiple forms of replication stress. These results are consistent with a recent study that found USP37-depleted cells are more sensitive to camptothecin (59). Although this study shows similar impacts as our own analyses of the viability of USP37-depleted cells, it is worth noting that RNAi-mediated knockdown likely leaves sufficient enzyme to carry out some of the functions of DUB, which may attenuate some phenotypes. In support of such a potential dosage effect, we find that stable reduction of USP37 levels achieved with shRNA provide similar impacts as does a more efficient, yet transient, reduction of enzyme levels resulting from siRNA. In addition, review of the shRNA and CRISPR screening data from Project Achilles *via* the DepMap portal (www.depmap.org) revealed that USP37 was found to be essential in 0.4% of cell lines (2 of 547) when depleted by shRNA, whereas 77% (621 of 808 cell lines) are sensitive to CRISPR-mediated loss. Notably, examining results of 372 cell lines that were analyzed in both the shRNA and CRISPR screens revealed nearly identical results with 75% of cells sensitive to CRISPR-mediated loss and 0.5% sensitive to depletion by shRNA. We expect that the use of CRISPR as a means to study the effects of USP37 loss will be informative. However, the severe effects of such loss currently hinder the generation of cells to perform these experiments. It will be important to develop a model that allows more complete loss of USP37 in order to gain further insight into its functions.

USP37 has been implicated in the regulation of DNA repair, primarily homologous recombination (51). Whereas defects in repair could contribute to the increased sensitivity to replication stress or camptothecin detected in the absence of USP37, several observations suggest that increased susceptibility to insults to the replication process underlies the increased number of γH2AX and 53BP1 foci and subsequent failure to proliferate in these cells. First, USP37 has been found to interact with the replication machinery, indicating a role in the process, which is supported by altered replication timing and fork dynamics in USP37-depleted cells (36, 37). Second, the concentration of APH used in our live cell proliferation and foci analyses studies results in moderate fork slowing that is not associated with the formation of double-strand breaks (55). Third, upon release from a thymidine-induced replication block, we observed an increase in 53BP1 foci in the absence of USP37 with γH2AX foci elevated similarly. Given that these foci were present in replicating cells, we interpret these foci to indicate stalled or malfunctioning forks. However, as the homologous recombination pathway is involved in the restart of stalled forks as well as the repair of collapsed forks, it is likely that USP37 deficiency leads to enhanced sensitivity by lowering the threshold for replication stress induction and impairing the mechanisms that allow for overcoming and recovering from challenges to the process. Future studies will determine the fate of replication forks and the nature of the lesions marked by γH2AX and 53BP1 in the absence of UPS37.

CHK1 activity is tightly regulated on multiple levels including the degradation of active CHK1 or of the upstream factor Claspin by the UPS (10, 11, 12, 13, 14, 15, 16, 17, 23, 24, 25, 26). Multiple DUBs regulate CLASPIN, whereas only ATAXIN-3, USP1, and USP7 have been implicated in regulation of CHK1 (11, 12, 16, 17, 19, 20, 21, 33). Intriguingly, regulation of CHK1 by these DUBs may be context specific. USP1 indirectly regulates CHK1 by limiting ubiquitination of Fanconi anemia complementation group D2 protein/I and/or proliferating cell nuclear antigen, which stimulates DNA damage–binding protein 1–dependent degradation of CHK1 (32). This mechanism may largely be important for types of damage that are dependent upon the Fanconi anemia pathway for repair, including that caused by mitomycin C. Similarly, USP7 regulates CHK1 most efficiently under conditions that activate ATM, which results in the stabilization of ZEB1, a factor that enhances the USP7–CHK1 interaction (30, 31). In contrast, ATAXIN-3 association with CHK1 is negatively regulated by DNA damage and replication stress (33). Given that UPS37 expression and activity peak during S phase, our findings suggest that USP37 regulates CHK1 during replication and, in particular, under conditions of replication stress. Increased replication stress, driven by oncogene activation, is inherent to cancer cells leading to an increased dependence on CHK1 and subsequently increased expression in cancers. Similar to CHK1, USP37 expression is elevated in multiple cancers. It will be interesting to determine whether the increased expression of USP37 in cancer reflects both an ability to drive replication as well as enhancing the ability to deal with replication stress caused by rapid cell cycle progression.

## Experimental procedures

### Cell lines

293T, HCT116, HeLa, MDA-MB-231, MDA-MB-468, MCF7, and U2OS cells were all obtained from the American Type Culture Collection. Cell lines are verified using the OSUCCC Genomics Shared Resource and routinely tested for mycoplasma. For reagents and materials, please refer to the Supporting Table.

### Cell culture

Cell lines were obtained from the American Type Culture Collection. 293T, H1299, HCT116, HeLa, MDA-MB-231, MDA-MB-468, MCF7, and U2OS cells were cultured in Dulbecco's modified Eagle's medium (Corning) supplemented with 10% fetal bovine serum (Seradigm) and 1% penicillin/streptomycin (GIBCO). All cells were incubated at 37 °C and 5% CO_2_. For synchronization *via* double thymidine block, cells were treated with 2 mM thymidine for 18 h and released into fresh media after washing with PBS. After 9 h, cells were again treated with 2 mM thymidine for 18 h and then released into S phase by washing with PBS.

### Transfections and treatments

siRNA transfections were performed using Lipofectamine RNAiMAX (Thermo Fisher Scientific) according to the manufacturer's protocol. Sequences of siRNAs used in this study are described in Table S1. Plasmid transfections were performed using Mirus TransIT-LT1 reagent (Mirus Bio) according to the manufacturer's protocol. CHX (Thermo Fisher Scientific) was used at a concentration of 100 μg/ml for the indicated times. APH, HU, and thymidine concentration and treatment duration are described in the legends to the figures.

### Molecular cloning

Complementary DNA encoding AURKB was cloned into pCS2+ MYC-DEST using Gateway Technology (Thermo Fisher Scientific).

### Immunoblotting

Cell extracts were generated in EBC buffer (50 mM Tris [pH 8.0], 120 mM NaCl, 0.5% Nonidet P-40, 1 mM DTT, and protease and phosphatase inhibitor tablets [Thermo Fisher Scientific]). Protein concentration was quantified by Pierce bicinchoninic acid assay (Thermo Fisher Scientific), and samples were prepared by boiling in Laemmli buffer for 5 min. Equal amounts of whole cell lysates were resolved by hand-cast SDS-PAGE and transferred to polyvinylidene difluoride membranes (Millipore). All blocking and primary antibody steps were performed in 5% nonfat dried milk diluted in Tris-buffered saline with Tween-20 (TBST) (137 mM NaCl, 2.7 mM KCl, 25 mM Tris, pH 7.4, and 1% Tween-20), except for phospho-CHK1, which as incubated in 5% bovine serum albumin (BSA) diluted in TBST. All primary antibody incubations were performed with shaking at room temperature (for epitope-tagged proteins) or at 4 °C for 16 h (for endogenous proteins). All secondary antibody incubations were performed with shaking at room temperature for 30 min in TBST + 0.02% SDS. Washing steps were performed using TBST, and protein bands were visualized using the LI-COR Odyssey CLx infrared imaging system (LI-COR Biosciences).

### Immunofluorescence, microscopy, and flow cytometry

Detection of DNA synthesis in proliferating cells was determined based on the incorporation of 5-ethynyl-2′-deoxyuridine (Click-IT EdU; Thermo Fisher Scientific) and its subsequent detection by a fluorescent azide through “click” chemistry per the manufacturer's instructions. In brief, cells were pulsed for 15 min with 10 μM EdU (Thermo Fisher Scientific) and fixed in 3.7% formaldehyde and washed in PBS prior to EdU labeling by click chemistry. For detection of DNA damage, U2OS or HeLa cells were seeded on glass coverslips and transfected with the indicated siRNAs. After 24 h, cells were treated overnight with vehicle or 200 nM APH. Cells were fixed and permeablized with 0.5% Triton X-100 in PBS, washed, and then blocked for 30 min at room temperature with 5% BSA in PBS. Cells were incubated with antibodies (1:500) in 5% BSA in PBS with Tween-20 (PBST) for 1 h at room temperature. After washing, the cells were incubated with Alexafluor secondary antibodies (1:500) in 5% BSA in PBST for 30 min at room temperature. DNA was counterstained with 1 μg/ml Hoechst 33342 and mounted with Fluoromount G (Thermo Fisher Scientific). Cells were imaged using a Leica DM5500B fluorescent microscope as described previously. Images were analyzed and foci quantified using FIJI software (60). For combined EdU and DNA damage analysis, EdU detection was performed first as per the manufacturer's instructions.

For flow cytometry, cells were incubated with the indicated siRNAs and harvested at the indicated times. For cell cycle progression studies, the cells were pulsed with EdU, as aforementioned, 48 h after transfection. The cells were then harvested at the indicated times. Cells were fixed in ice-cold 70% ethanol and permeabilized in 0.1% Triton X-100 for 15 min on ice. EdU was visualized as aforementioned, and the cells were incubated on with 200 μg/ml RNAse A and 50 μg/ml propidium iodide for 30 min at 37 °C. Cells were then analyzed on a Becton Dickinson FacsCalibur instrument and analyzed with FlowJo software (FlowJo, LLC).

### Immunoprecipitations

Cells were transfected with the indicated plasmids and lysed in EBC buffer 48 h after transfection. About 1 μg of the indicated antibody was mixed with equal volumes of lysate at 4 °C overnight. Magnetic Protein A and Protein G beads (Thermo Fisher Scientific) were then added and incubated at 4 °C for an additional 30 min. Beads were washed three times with EBC buffer, and proteins were eluted by boiling beads in Laemmli buffer for 5 min and visualized by immunoblotting.

### *In vitro* binding assay

pGEX6P1-USP37 or pGEX6P1-CDH1 was transformed into *Escherichia coli* BL21(DE3) cells. For expression of UPS37, bacteria were inoculated into a 20-ml culture in Overnight Express media and grown for 8 h at 37 °C. The culture was used to inoculate two 1 l cultures and grown for 15 h at 20 °C. The absorbance at 600 nm was checked, and the cells were grown for an additional 4 h. The absorbance at 600 nm was stable, and the cells were incubated for an additional 8 h and harvested. For expression of GST-CDH1, bacteria were grown in standard LB media to an absorbance of 0.5 at 600 nm and induced to express by addition of 0.1 mM IPTG and incubated with shaking at 18 °C for 16 h. Bacterial pellets were frozen in liquid nitrogen. Thawed cells were resuspended in 60 ml lysis buffer (50 mM Tris, pH 8, 500 mM NaCl, 0.5% Triton X-100, and protease inhibitor tablets). GST fusions were purified from crude lysate on glutathione sepharose (GE Lifesciences) following the manufacturer's protocol. Following elution, proteins were buffer exchanged (20 mM Hepes, pH 7.7, 100 mM KCl, 1 mM DTT, and 5% glycerol) using Slide-a-lyzer dialysis units (Thermo Fisher Scientific) following the manufacturer's protocols. MYC-CHK1 and MYC-AURKB were generated in a rabbit reticulocyte-coupled transcription/translation system (Promega) following the vendor's protocol.

For binding assays, 2 μg of GST or 100 ng of GST-USP37 were mixed with 10 μl of *in vitro* translated proteins on ice for 1 h. Then, the proteins were diluted with TBST and mixed glutathione beads for 1 h at 4 °C. Following four washes with TBST, protein complexes were eluted in Laemmli buffer and assessed by immunoblotting.

### S-phase progression assays

U2OS cells were seeded on glass coverslips and transfected with the indicated siRNAs. After 24 h, the cells were pulse labeled with 10 μM IdU for 30 min and placed into fresh media. At 8 h post-IdU, the cells were pulse labeled with 10 μM CldU for 30 min. The cells were then fixed in 70% ethanol for 10 min at room temperature. After permeabilization in methanol, chromatin was denatured with 1.5 N HCl for 30 min, followed by blocking with BSA in PBST (PBS + 0.01% Tween-20). The coverslips were then incubated with sequentially with rat anti-BrdU, antirat secondary, mouse anti-BrdU, and antimouse secondary antibodies. All antibody incubations were followed by three washes in PBST. The DNA was counterstained with Hoechst 33342, and coverslips were mounted with Flouromount G.

### Proliferation assays

HCT116 H2B-GFP HeLa, H1299, MCF7, and MDA-MB-468 cells were seeded in 96-well plates. The cells were then transfected with the indicated siRNAs and incubated for 6 h before the addition of drug, where indicated. For rescue experiments, cells were seeded in 6-cm dishes and transfected with the indicated plasmids. Twenty four hours later, cells were processed as aforementioned. Growth of triplicate samples was monitored by phase-contrast imaging or GFP count (HCT116 H2B-GFP cells) using the IncuCYTE Zoom (Essen Bioscience) system. Cells were imaged every 4 h for 72 h, and growth was determined as fold increase of GFP+ cells or percent phase confluence at the start of imaging.

### Colony formation assay

HCT116, HeLa, H1299, MCF7, or U2OS cells were transfected with the indicated siRNA, shRNA, or plasmid constructs, then 24 h later, seeded in triplicate for each condition in 6-well dishes. After 24 h, cells were treated with 1 mM HU, 200 mM thymidine, or 2.5 μg/ml APH for 18 h. The cells were washed and given fresh media. Colonies were fixed and stained in 0.5% w/v crystal violet and 25% methanol after 10 to 14 days.

### *In vivo* deubiquitination assay

293T cells were transfected with the indicated plasmids and siRNAs. After 48 h, the cells were treated with 10 mM HU and 100 μM MG132 for 4 h and then harvested. For Figure 7*A*, 90% of the cell suspension was lysed in 6 M guanidine–HCl, 100 mM Na_2_HP0_4_–NaH_2_P0_4_, 10 mM Tris–HCl, pH 8.0, 5 mM imidazole, 10 mM β-mercaptoethanol, and 6xHIS-ubiquitinated proteins were captured on nickel–nitrilotriacetic acid resin (Thermo Fisher Scientific) for 4 h at room temperature. The beads were washed sequentially in lysis buffer (without imidazole), buffer A, pH 8 (8 M urea, 100 mM Na_2_HP0_4_–NaH_2_P0_4_, 10 mM Tris–HCl ,pH 8.0, and 10 mM β-mercaptoethanol), buffer A pH 6.3 + 0.2% Triton X-100, and buffer A pH 6.3 + 0.1% Triton X-100, and eluted by boiling in Laemmli buffer containing 200 mM imidazole. About 10% of the sample was used to prepare inputs. For Figure 7, *B* and *C*, lysates were generated in 1× EBC buffer without DTT and including 50 mM *n*-ethylmaleimide. SDS was added to lysates at a final concentration of 1%, and lysates were boiled for 10 min. The lysates were then subjected to nickel–nitrilotriacetic acid pulldown as aforementioned and washed with EBC + 1% SDS and eluted as aforementioned. Pull-down eluates and inputs were separated on SDS-PAGE gels and analyzed by immunoblot.

### Protein quantification and stability

Protein bands were measured with Image Studio (LI-COR Bioscience), using median background subtraction. For determination of phospho-CHK1 levels, signal intensity was normalized to the signal of the unmodified CHK1 in the same lane. For total CHK1 levels, signal intensity was normalized to the signal of ACTIN in the same lane. To analyze CHK1 stability, the protein level at time 0 was set to 1, and the fraction remaining was determined for subsequent time points. The protein half-life was determined using GraphPad Prism (GraphPad Software, Inc) to perform best-fit nonlinear regression.

### Zebrafish studies

Fish care and animal work were carried out in compliance with the National Institutes of Health (NIH) guidelines for animal care and approved by the National Cancer Institute at Frederick Animal Care and Use committee (study proposal: 17-416). Zebrafish used in these studies were of wildtype AB.

Knockdowns for Usp37 were carried out using the following translation blocking morpholino obtained from GENETOOLS: USP37 5′-CCATCAGGGCGAAGATCCTCCACAA-3′.

Embryos were injected with 250 μM of translation blocking morpholino at the one-cell stage using a microinjector PLI-90 (Harvard Apparatus). For HU treatments, embryos were treated with 125 mM at 24hpf for 16 h and examined at 72hpf. For acridine orange staining, embryos were placed at 24hpf in E3 medium with phenylthiourea as a pigment clearing method in the presence or the absence of 125 mM HU. At 72 hpf, embryos were incubated for 1 h in acridine orange (5 μg/ml) followed by three washes in E3 medium and imaged live in anesthetic reagent (MS-222) using a 4× water immersion objective (0.13 numerical aperture) and a Nikon Eclipse Ni-E upright fluorescence microscope equipped with a DS-Ri2 camera.

For expression analysis, embryos were dechorionated and homogenized in lysis buffer (20 mM Tris at pH 8, 137 mM NaCl, 10% glycerol, and 1% Triton X-100) with protease inhibitor cocktail (Roche), centrifuged for 10 min at 13,000 rpm, and supernatants were boiled in sample buffer.

### Statistical analysis

Statistical analyses were performed with GraphPad Prism Software using an unpaired Student's *t* test one- or two-way ANOVA with post-tests, as indicated.

## Data availability

All data are presented in the article.

## Supporting information

This article contains supporting information (34, 35, 46).

## Conflict of interest

The authors declare that they have no conflicts of interest with the contents of this article.
