# Supplementary material for: The deubiquitinating enzyme USP37 enhances CHK1 activity to promote the cellular response to replication stress
Source: J Biol Chem. 2021 Sep 10;297(4):101184. doi: 10.1016/j.jbc.2021.101184 (PMC8487067; doi:10.1016/j.jbc.2021.101184)
Supplement: Supplemental Figures S1–S5 and Table S1 [file mmc1.pdf]

Supplemental figures

Stromberg et al, Figure S1

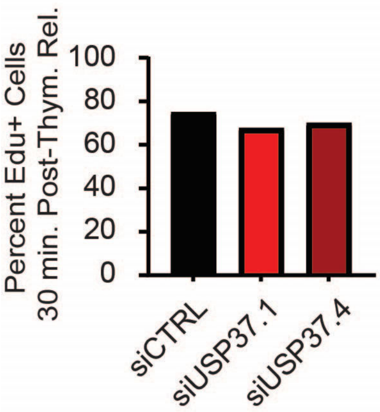

Figure S1. USP37-depleted cells enter s-phase with control-depleted cells after release from thymidine, related to Figure 3**.** HeLa cells, treated as in Figure 1E were pulsed with EdU 30 minutes after release from the second thymidine block. The number of cells in S-phase (EdU+) in each population are indicated.

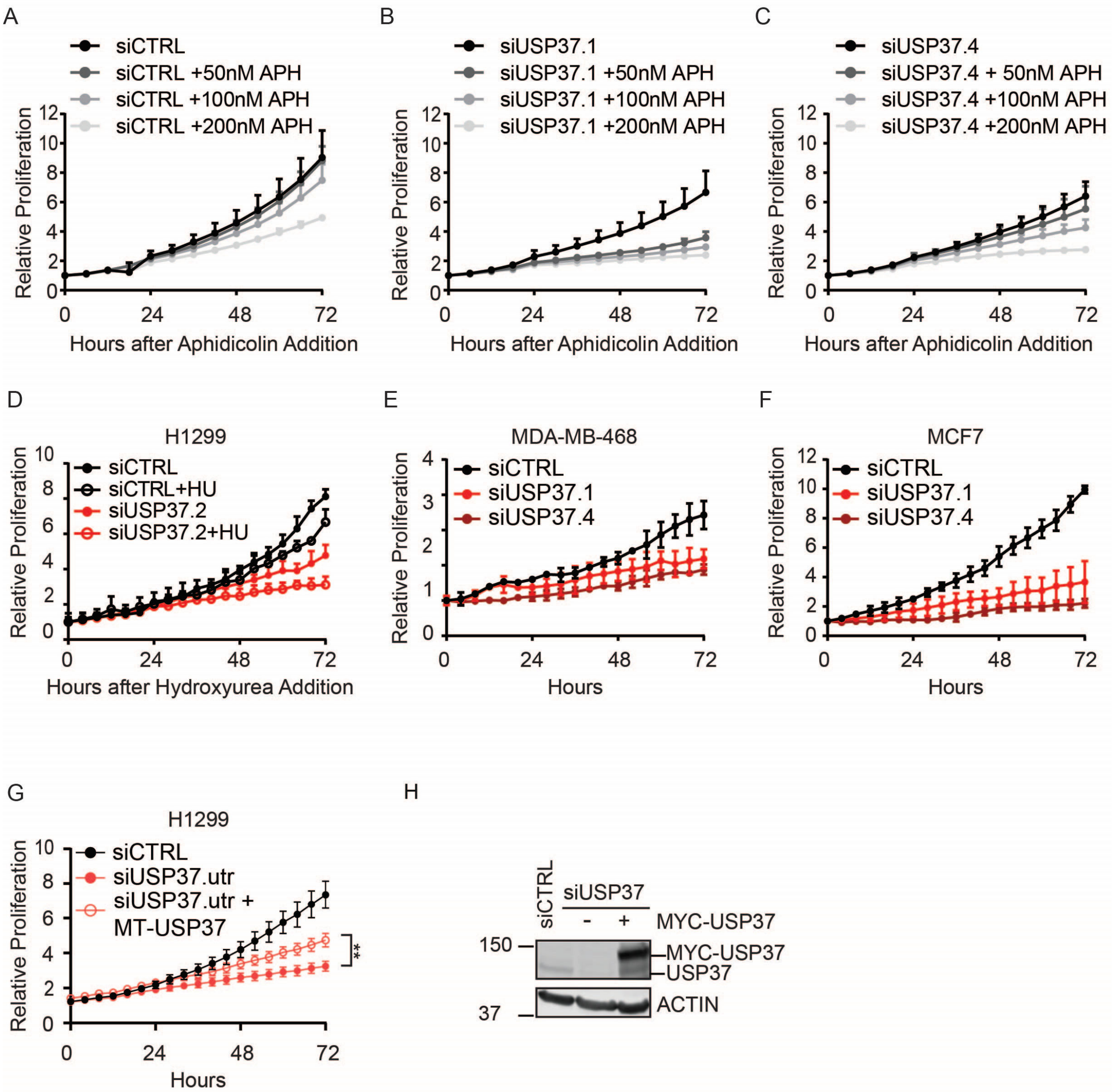

Figure S2. USP37 promotes short-term proliferation and tolerance of replication stress, related to Figure 4. **A.** Data from two independent experiments, performed in triplicate. Mean and standard deviation are indicated. **B.** Cell proliferation (normalized to t=0) was analyzed in HCT116-H2BGFP cells transfected with the indicated USP37-targeting siRNA (siUSP37.1) ± the indicated concentrations of APH. Data from two independent experiments, performed in triplicate. Mean and standard deviation are indicated. **C.** Cell proliferation (normalized to t=0) was analyzed in HCT116-H2BGFP cells transfected with the indicated USP37-targeting siRNA (siUSP37.4) ± the indicated concentrations of APH. Data from two independent experiments, performed in triplicate. Mean and standard deviation are indicated. **D.** Cell proliferation (normalized to t=0) was analyzed in H1299 cells transfected with the indicated siRNAs ± 100 μM HU. Data is

from an experiment, performed in triplicate. Mean and standard deviation are indicated. **E.** Cell proliferation (normalized to t=0) was analyzed in MDA-MB-468 cells transfected with the indicated siRNAs. Data is from an experiment, performed in triplicate. Mean and standard deviation are indicated. **F.** Cell proliferation (normalized to t=0) was analyzed in MCF7 cells transfected with the indicated siRNAs. Data is from an experiment, performed in triplicate. Mean and standard deviation are indicated**. G.** Cell proliferation (normalized to t=0) was analyzed in H1299 cells transfected with vector or MYC-USP37 and the indicated siRNAs ± 100 μM HU. Data from three independent experiments, performed in triplicate. Mean and standard error of the mean are indicated. Data were analyzed by two-way ANOVA with Holm-Sidak post-test. **, $p<0.01$. **H.** Lysates from cells treated as in G were analyzed by immunoblot with the indicated antibodies.

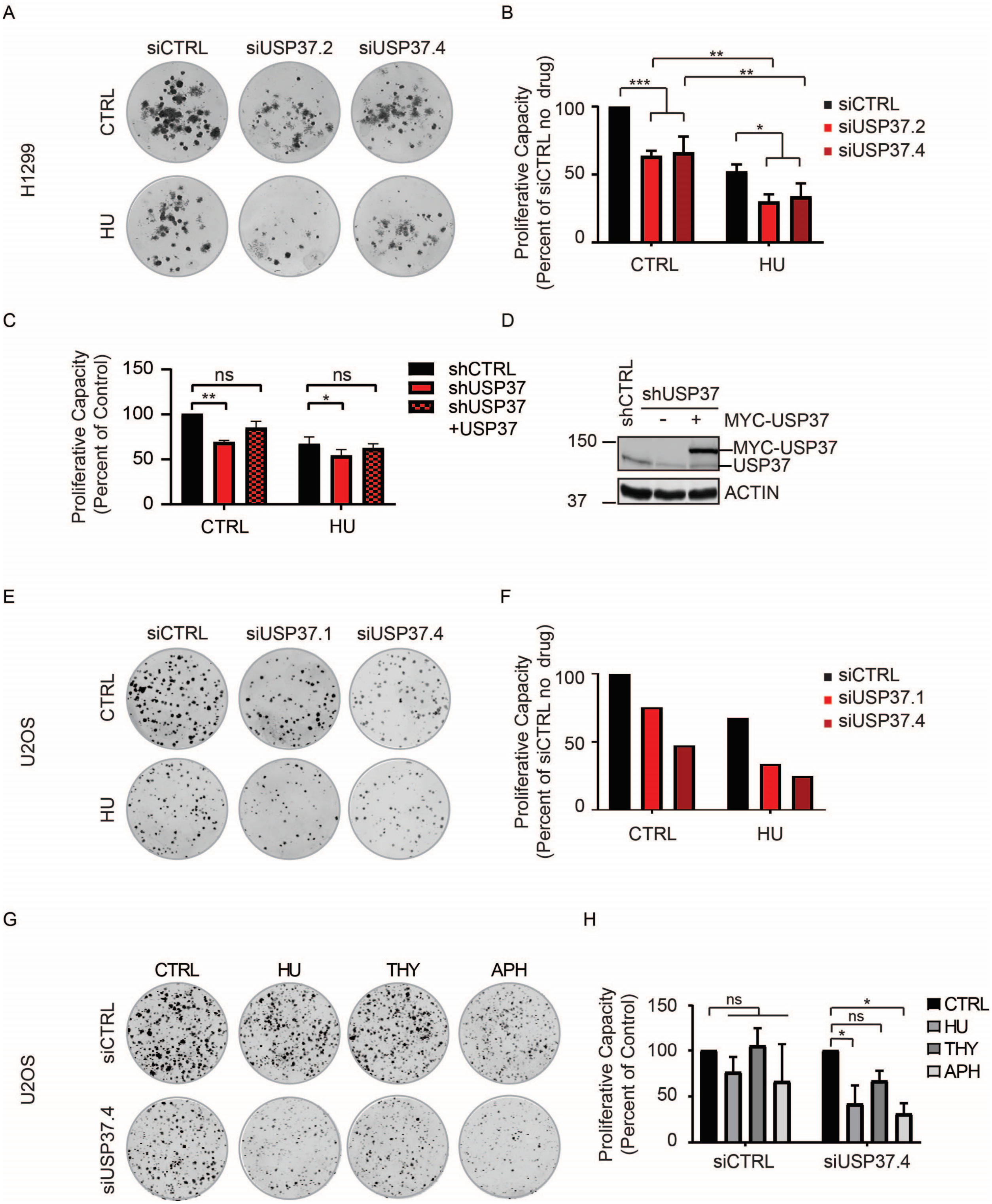

Figure S3. USP37 promotes colony formation and tolerance of replication stress, related to Figure 4. **A.** Colony formation of H1299 cells transfected with the indicated siRNAs ± treatment with 1 mM HU for 18 hours. **B.** Quantification of data as in (A), normalized to control, no drug. Data from three independent experiments, performed in triplicate. Mean and standard deviation are indicated. Data were analyzed by

two-way ANOVA with Tukey's post-test; *,p<0.05, **,p<0.01, ***, p<0.001 **C.** H1299 cells were transfected with vector control or MT-USP37 plasmids and the indicated shRNAs and then treated as in B. Data from three independent experiments, performed in triplicate. Mean and standard deviation are indicated. Data were analyzed by two-way ANOVA with Holm-Sidak post-test. *, p<0.05, **, p<0.01. **E.** Colony formation of U2OS cells transfected with the indicated siRNAs ± treatment with 1 mM HU for 18 hours. **F.** Quantification of data as in (C), normalized to control, no drug. Data from an experiment, performed in triplicate. Mean and standard deviation are indicated. **G.** Colony formation of U2OS cells transfected with the indicated siRNAs ± treatment with 1 mM HU, 200 mM, THY, or 7 µM APH for 18 hours. **H.** Quantification of data as in (F), normalized to control conditions for each siRNA. Data from three independent experiments, performed in triplicate. Mean and standard deviation are indicated. Data were analyzed by two-way ANOVA with Dunnet's post-test; *,p<0.05.

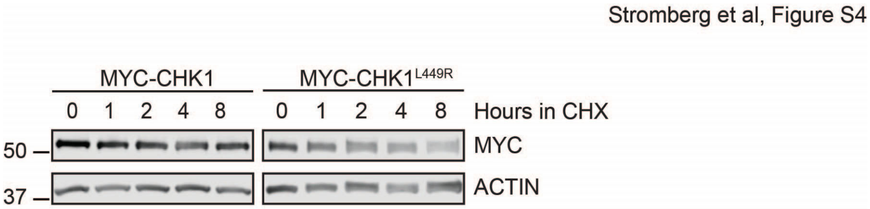

Figure S4. 293T cells were transfected with plasmids encoding MYC-CHK1 or constitutively active MYC-CHK1^{L449R} and analyzed by immunoblot after treatment with 100 µg/ml cycloheximide (CHX).

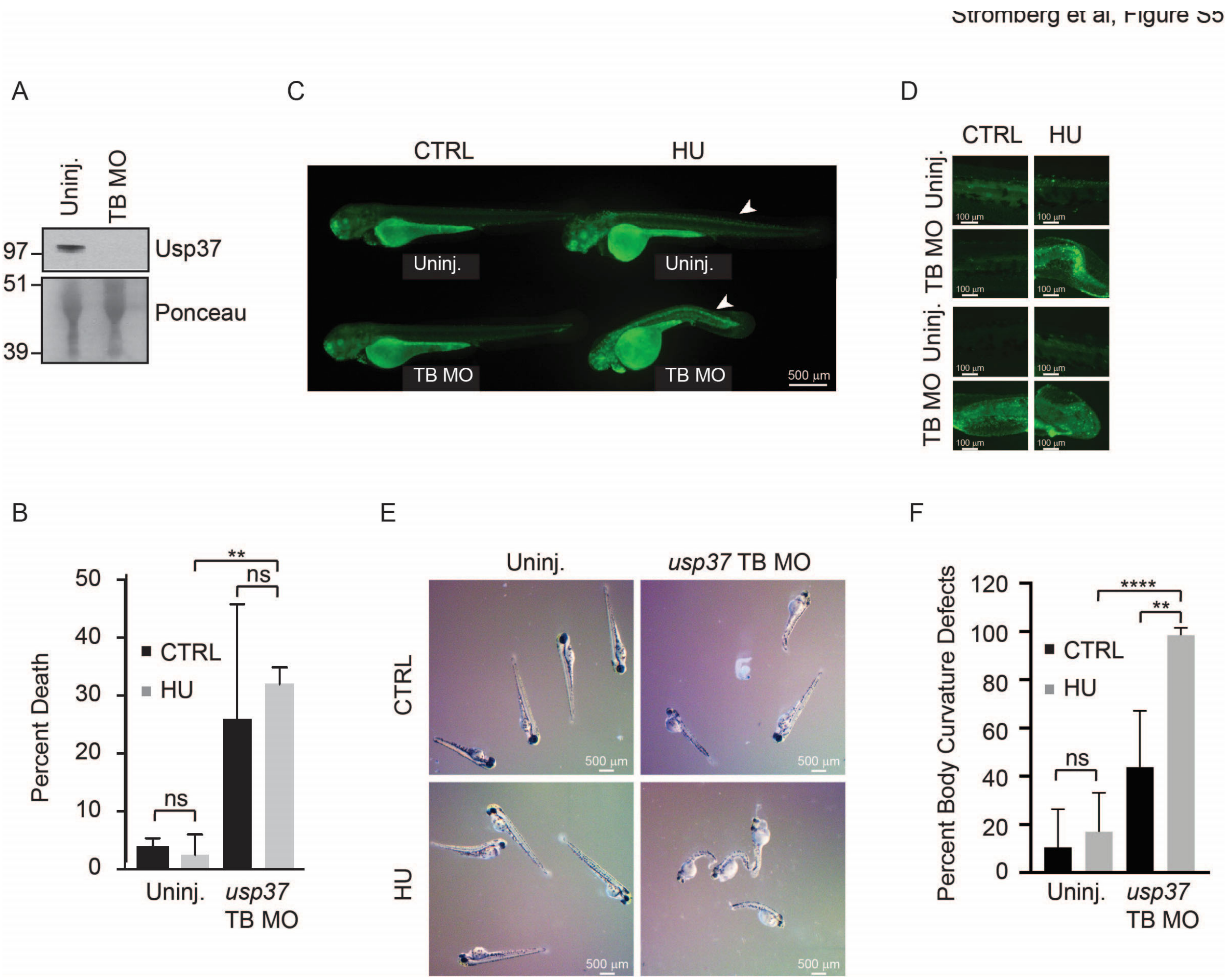

Figure S5. USP37 promotes growth tolerance of replication stress in zebrafish embryos, related to Figure 4. **A.** USP37 protein expression was examined by immunoblot after injection with a USP37 translation-blocking morpholino (TB MO). **B.** Analysis of death in 72 hpf embryos uninjected or injected with USP37 TB MO and untreated or treated with 125 mM hydroxyurea for 16 hours at 24hpf. These data are from 2 independent experiments with total numbers of fish counted as follows; Uninj. -HU, n=71; Uninj. +HU, n=90; TB –HU, n=47; TB +HU, n=51. One-way ANOVA was applied to obtain p values. ** p<0.01 **C.** Fish, as in (B), treated with 125 mM HU for 24 hours and stained with acridine orange to visualize apoptotic cells. Arrowheads indicate the regions of increased apoptosis visualized in the tail. **D.** Representative images of tails of fish from two different groups (upper and lower panels), examined as in (C), from 3 independent experiments. **E.** Representative brightfield images of live 72 hpf embryos uninjected or injected with USP37 TB MO ± HU. **F.** Quantification of body curvature defects in embryos as in (E). Mean and SD from 4 independent experiments with total numbers of fish counted as follows; Uninj. -HU, n=62; Uninj.

+HU, n=73; TB –HU, n=38; TB +HU, n=28. One-way ANOVA was applied to obtain p values. ** $p< 0.005$, **** $p<0.0001$

Table S1. Reagents and materials

Reagent or resource	Source	Identifier
**Antibodies**		
53BP1	BD Biosciences	Cat# 612522, RRID:AB_2206766
BrdU	ThermoFisher	Cat# MA1-82088, RRID:AB_927214
BrdU	BD Biosciences	Cat# 347580, RRID:AB_400327
CHK1	Santa Cruz	Cat# sc-8408; RRID:AB_627257
CHK1 phospho-S345	CST	Cat# 2348S; RRID:AB_331212
Cyclin A1	Santa Cruz	Cat# sc-15383, RRID:AB_2071995
Donkey anti-Mouse IgG (H+L) Highly Cross-Adsorbed Secondary Antibody, Alexa Fluor 488	ThermoFisher	Cat# A-21202
Donkey anti-Rabbit IgG (H+L) Highly Cross-Adsorbed Secondary Antibody, Alexa Fluor 488	ThermoFisher	Cat# A-21206
Donkey anti-Rat IgG (H+L) Highly Cross-Adsorbed Secondary Anti-body, Alexa Fluor 594	ThermoFisher	Cat# A-21209
FLAG M2	Sigma-Aldrich	Cat# F1804; RRID: AB_262044
HA	BioLegend	Cat# 901502, RRID:AB_2565007
HIS	Millipore-Sigma	Cat# H1029, RRID:AB_260015
IRDye® 680RD Donkey anti-Mouse IgG Secondary Antibody	LI-COR Biosciences	Cat# 926-68072
IRDye® 680RD Donkey anti-Rabbit IgG Secondary Antibody	LI-COR Biosciences	Cat# 926-68073
IRDye® 680RD Goat anti-Rat IgG Secondary Antibody	LI-COR Biosciences	Cat# 926-68076
IRDye® 800CW Donkey anti-Mouse IgG Secondary Antibody	LI-COR Biosciences	Cat# 926-32212
IRDye® 800CW Donkey anti-Rabbit IgG Secondary Antibody	LI-COR Biosciences	Cat# 926-32213
IRDye® 800CW Goat anti-Rat IgG Secondary Antibody	LI-COR Biosciences	Cat# 926-32219

MYC	Santa Cruz	Cat# sc-40;H RRID: AB_627268
MYC	ThermoFisher	Cat# 13-2500, RRID:AB_2533008
MYC	CCF Hybridoma Core	9E10
Phospho-Histone H3 S10	CST	Cat# 3377; RRID: AB_1549592
USP37	Huang et al	N/A
β-ACTIN	Millipore-Sigma	Cat# A3854, RRID:AB_262011
γH2AX	Millipore-Sigma	Cat# 16-193, RRID:AB_310795

Bacterial Strains

Escherichia coli BL21(DE3) Chemically Competent Cells	Thermo Fisher	Cat# C600003
Escherichia coli NEB 5-alpha Chemically Competent Cells	NEB	Cat# C2987I
Escherichia coli NEB Stable Chemically Competent Cells	NEB	Cat# C3040I

Chemicals, Peptides, and Recombinant Proteins

5-Chloro-2′-deoxyuridine	MilliporeSigma	Cat# C6891
5-Iodo-2′-deoxyuridine	MilliporeSigma	Cat# I7125
Acridine Orange	MilliporeSigma	Cat# A6014
Aphidicolin	MilliporeSigma	Cat# 178273
Cycloheximide	ThermoFisher	Cat# AC357420010
Dynabeads	ThermoFisher	Cat# 10004D
Dynabeads Protein G	ThermoFisher	Cat# 10003D
Fluoromount-G	ThermoFisher	Cat# 00-4958-02
Glutathione Agarose Resin	MilliporeSigma	Cat# GE17-0756
GST-CDH1	Pal, et al. 2019	N/A
GST-USP37	Huang, et al. 2011	N/A
HIS-Select Nickel Affinity Gel	MilliporeSigma	Cat# P6611
Hydroxyurea	MilliporeSigma	Cat# H8627M
Lipofectamine RNAiMAX	Thermo Fisher	Cat# 13778150
MG132	R&D Systems	Cat# I-130
Nocodazole	MilliporeSigma	Cat# M1404
Odyssey® One-Color Protein Molecular Weight Marker	LI-COR Biosciences	Cat# 928-40000
Paclitaxel	MilliporeSigma	Cat# 580556
Pierce Anti-c-Myc Magnetic Beads	ThermoFisher	Cat# 88842
Propidium Iodide	MilliporeSigma	Cat# P4864

RNase A	MilliporeSigma	Cat# R6513
Thymidine	MilliporeSigma	Cat# T1895
TransIT-LT1 Reagent	Mirus Bio	Cat# MIR 2304

Critical Commercial Assays

Click-IT EdU Imaging Kit	ThermoFisher	Cat# C10340, C10639
Pierce BCA Protein Assay Kit	ThermoFisher	Cat# 23225
TnT® Quick Coupled Transcription/ Translation System	Promega	Cat# L2080
LookOut Mycoplasma PCR Detection Kit	MilliporeSigma	Cat# MP0035

Experimental Models: Cell Lines

293T	ATCC	Cat# CRL-3216; RRID: CVCL_0063
H1299	ATCC (gift of A. Strohecker)	CRL-5803, RRID:CVCL_0060
HCT116		
HeLa	ATCC	Cat# CCL-2; RRID: CVCL_0030
MCF7	ATCC	Cat# HTB-22; RRID: CVCL_0031
MDA-MB-468	ATCC	Cat# HTB-132; RRID: CVCL_0419
U2OS	ATCC	HTB-96, RRID:CVCL_0042

Experimental Models: Organisms/Strains

Zebrafish		

Oligonucleotides

ON-TARGETplus USP37 siRNA CAAAAGAGCUACCGAGUUA	Dharmacon	J-006085-06-0010
ON-TARGETplus USP37 siRNA CCAAGGAUAUUUCAGCUAA	Dharmacon	J-006085-07-0010
ON-TARGETplus USP37 siRNA GAAUAAAGUCAGCCUAGUA	Dharmacon	J-006085-09-0010
DsiRNA USP37 TTAACCACCTATGTAATCTCATGTT	IDT	hs.Ri.USP37.13.1
Morpholino Oligo	GENETOOLS, LLC	N/A

Recombinant DNA

pCDNA5-6xHIS-Ubiquitin	Burrows, et al. 2012	
pCS2-FLAG		
pCS2-MYC-AURORA B	This study	

pCS2-MYC-CHK1	Pal, et al. 2019	
pCS2-MYC-CHK1 L449R	Pal, et al. 2019	
pCS2-MYC-USP37	Burrows, et al. 2012	
pCS2-TAP-USP37	Huang, et al. 2011	
pCS2-TAP-USP37 C350A	Huang, et al. 2011	
pGEX6P1	GE Lifesciences	Cat# 28954648
pGEX6P1-CDH1	Pal, et al.	
pGEX6P1-USP37	Burrows, et al. 2012	
pLKO.1 shNonT	MilliporeSigma	Cat# SHC016
pLKO.1 USP37 3'UTR	MilliporeSigma	TRCN0000004569
**Software and Algorithms**		
FIJI		https://imagej.net/Fiji
FlowJo X	FlowJo LLC	https://www.flowjo.com
Prism 8	GraphPad	https://www.graphpad.com
CellProfiler	BROAD Institute	https://cellprofiler.org/
